# Inter-domain distance analysis reveals conformational changes in the Keap1-Nrf2 stress sensor system

**DOI:** 10.1016/j.redox.2026.104379

**Published:** 2026-09-01

**Authors:** Ritsumi Saito, Takeshi Kawabata, Seisuke Yamashita, Keisuke Oki, Takashi Fujii, Hiroki Kawauchi, Yoshiaki Doi, Machiko Irie, Takuya Torizawa, Kengo Kinoshita, Masayuki Yamamoto, Seizo Koshiba

**Affiliations:** a The Advanced Research Center for Innovations in Next-Generation Medicine (INGEM), Tohoku University, Sendai, Miyagi, Japan; b Tohoku Medical Megabank Organization, Tohoku University, Sendai, Miyagi, Japan; c Graduate School of Information Sciences, Tohoku University, Sendai, Miyagi, Japan; d Research Division, Chugai Pharmaceutical Co., Ltd., Kanagawa, Japan

**Keywords:** Keap1, Nrf2, cryo-EM, Protein-protein interaction inhibitor

## Abstract

The Keap1-Nrf2 system plays a central role in cellular defense against oxidative stress. Structural information on full-length Keap1 is essential for understanding the molecular basis of this regulation. However, its overall architecture has remained elusive due to pronounced conformational flexibility. In this study, we performed single-particle cryo-electron microscopy (cryo-EM) analysis of full-length Keap1 and found multiple particle conformations accompanied by severe preferred orientation in vitrified ice. To address these problems, we developed an analytical system that focuses on measuring the inter-domain distance between the two DC domains of the Keap1 homodimer. Using this approach, we identified the change of inter-domain distance distributions of Keap1 induced by Nrf2 binding or its inhibition, suggesting that these conformational changes are associated with Nrf2 regulation. Furthermore, through integration of this system with Keap1 deletion mutants, three flexible regions within Keap1 are found to contribute substantially to the conformational flexibility of the Keap1 homodimer. Together, these findings provide structural insights into the dynamic changes of the Keap1-Nrf2 system that would contribute to the development of Keap1-targeted therapeutics.

## Highlights

• Cryo-EM analysis indicates conformational diversity in full-length Keap1.
• A cryo-EM–based system quantitatively evaluates Keap1 domain-distance diversity.
• Nrf2 binding and a protein-protein interaction inhibitor alter Keap1 conformation.
• Three flexible regions contribute to Keap1 structural flexibility.

## Graphical abstract

**Figure:**
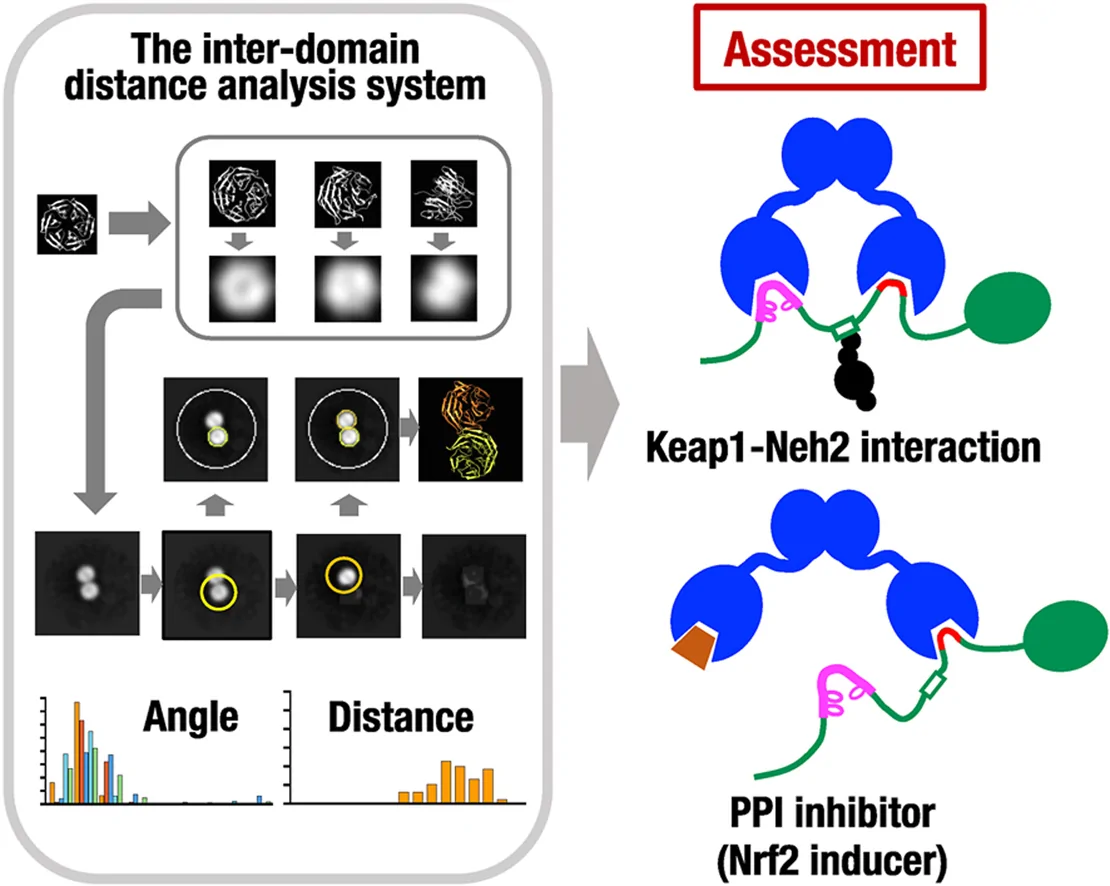

## 1 Introduction

Living organisms possess defense mechanisms against internal and environmental stress, and the transcription factor Nrf2 (NF-E2-related factor 2) plays a central role by inducing the expression of cytoprotective genes [[1], [2], [3]]. Keap1 (Kelch-like ECH-associated-protein 1), an adaptor protein of the Cul3-based ubiquitin E3 ligase complex, constitutively ubiquitinates Nrf2 and targets it for proteasomal degradation [[4], [5], [6], [7], [8]]. Modification of reactive stress-sensor cysteine residues within Keap1 by electrophiles or reactive oxygen species decreases its ubiquitin ligase activity, resulting in the stabilization of Nrf2. Stabilized Nrf2 then translocates to the nucleus, where it induces the expression of a battery of cytoprotective genes [[9], [10], [11], [12]]. Studies of the Keap1-Nrf2 regulatory system have revealed that Keap1 is a critical drug target for protection against oxidative stress and toxic chemicals. Therefore, a comprehensive understanding of the molecular mechanisms underlying the function and regulation of the Keap1-Nrf2 system will contribute to the prevention and development of therapeutic strategies for numerous chronic inflammatory diseases and cancers.

Keap1 is a 69 kDa protein composed of five distinct regions: NTR (N-terminal region), BTB (broad complex, tramtrack and bric-a-brac) domain, IVR (intervening region), DC (double glycine repeat or Kelch plus C-terminal) domain, and CTR (C-terminal region). The BTB domain mediates homodimerization [13]. The DC domain adopts a β-propeller structure and contains the binding site for Nrf2. The two DC domains of the Keap1 homodimer interact individually with the low-affinity DLGex and high-affinity ETGE motifs within the Neh2 domain of Nrf2 [[14], [15], [16], [17], [18]]. In addition, the BTB domain, IVR, and CTR contain multiple cysteine sensors that respond to oxidative and electrophilic stresses [11,12,[19], [20], [21], [22], [23]].

Structural information on Keap1 is a prerequisite for a comprehensive understanding of the Keap1-Nrf2 system. Although crystal structures of partial regions of Keap1 have been reported [16,[24], [25], [26]], no full-length crystal structure is currently available. A previous negative-staining electron microscopy study of full-length mouse Keap1 reported a low-resolution reconstruction at 24 Å resolution [27], consisting of two spherical bodies and a forked-stem structure via thin linkers. The estimated distance between the two globular domains was approximately 80 Å, while minor populations with both longer and shorter inter-domain distances were also observed, indicating the presence of flexible regions in Keap1. Owing to this structural flexibility, high-resolution structural determination of full-length Keap1 remains to be clarified.

In recent years, cryo-electron microscopy (cryo-EM) has been developed [28,29]. Compared with X-ray crystallography, cryo-EM does not require crystallization and enables structure determination under near-native, hydrated conditions, even when several conformational states coexist. Based on these advantages, we recognized that cryo-EM is suited for analyzing the Keap1 structure. Accordingly, we have been challenging the cryo-EM analysis to investigate the full-length structure of Keap1.

Our initial analysis revealed that Keap1 adopts multiple conformations and exhibits severe preferred orientation in vitrified ice. Therefore, in this study, we developed a single-particle analysis to obtain 2D class-average images and fit partial atomic models, which allowed us to quantitatively assess conformational distributions of Keap1 protein. Through focusing on the inter-domain distance between the two DC domains of the Keap1 homodimer, we successfully evaluated quantitatively the distribution of inter-domain distances. Here, we report the presence of ligand-dependent changes induced by Nrf2 binding and its release by a Keap1-Neh2 interaction inhibitor. Furthermore, similar analysis utilizing Keap1 deletion mutants demonstrates that three flexible regions within Keap1 substantially contribute to overall conformational mobility. Together, these findings support the Keap1-Nrf2 two-site binding Hinge-Latch model [13,15,16,18,30] and provide important insights into the structural basis of Keap1-Nrf2 regulation.

## 2 Results

### 2.1 Cryo-EM system for analyzing inter-DC-domain distances of Keap1

**Fig. 1:**
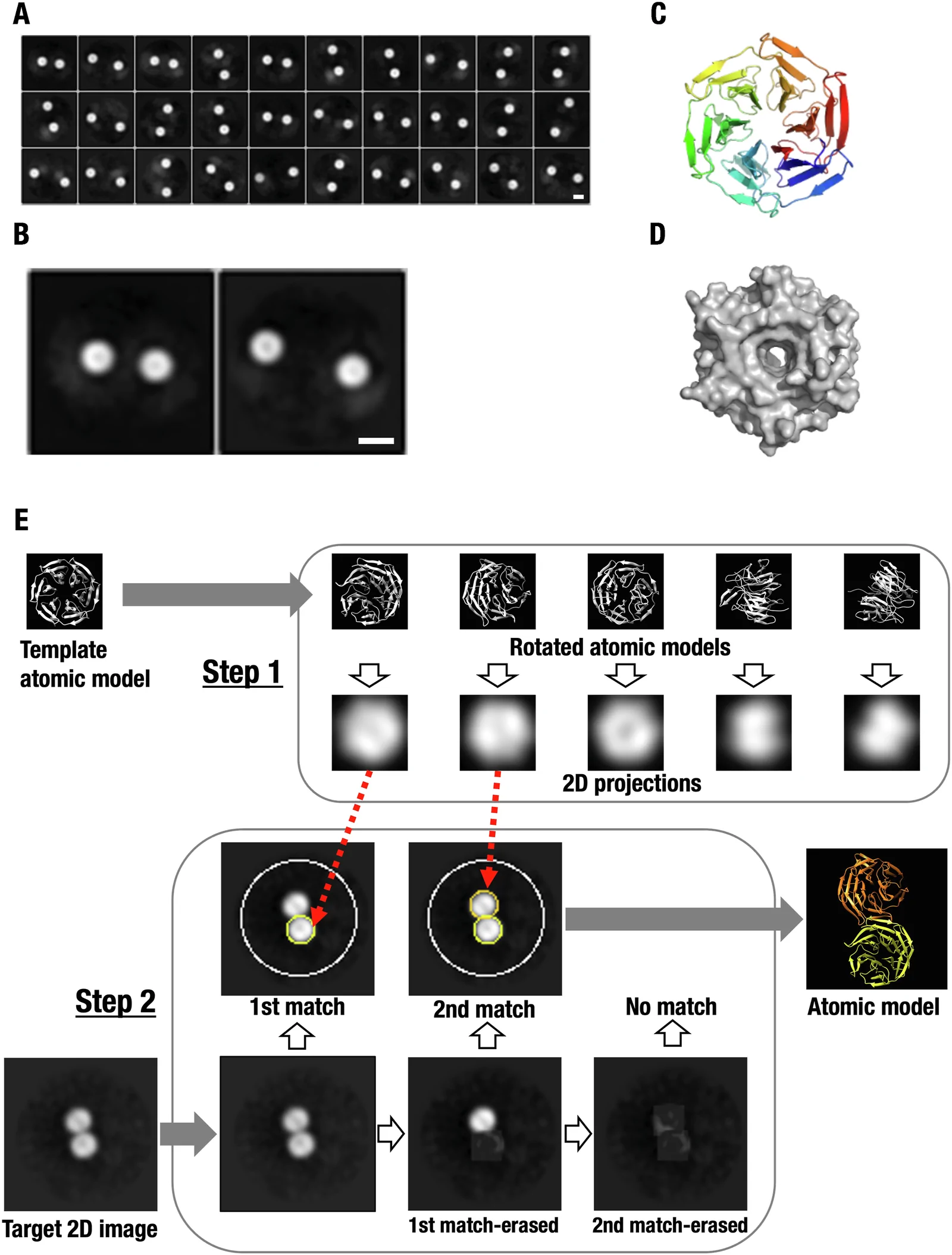
**Cryo-EM system for analyzing inter-DC-domain distances of Keap1.** (A) Representative 2D class averages of the full-length Keap1 homodimer. The white spheres indicate the density for a DC domain of Keap1, which shows a strong preferred orientation. The density for the remaining domains of Keap1 is weak or absent, presumably due to the structural flexibility of Keap1. Scale bar (bottom right): 50 Å. (B) Representative 2D class averages demonstrating the diversity of the inter-DC domain distances. The DC domain density exhibits a donut-shaped appearance, with a low-density region visible at its center. Scale bar (bottom right): 50 Å. (C), (D) Crystal structure of the DC (Kelch and C-terminus) domain of human Keap1 (PDB ID: 1U6D) shown as a ribbon model (C) and a surface model (D). (E) An outline of the template matching of 3D atomic models on 2D image. The Step 1 is for the generation of the projected 2D template images from rotated template 3D atomic models. The Step 2 is the matching the template images on the target image by exhaustively searching over all possible pixel-wise translations. The matching step is repeated until no more templates are detected. Before matching additional templates, the density of the previously template-matched region in the target image was erased.

Keap1 forms a homodimer through its BTB domain [31]. A previous negative-stain electron microscopy study suggested that the molecule adopts a cherry-bob-like shape, in which two DC domains are jointed via a BTB domain stem [27]. Therefore, it was expected that the cryo-EM data would contain projections of whole homodimers existing with all orientations within the ice. However, the 2D class averages of cryo-EM data of Keap1 alone showed only images of two circular domains positioned on a plane, while images of other domains seemed to be averaged out or blurred away (Fig. 1A). Moreover, the distance between the two spherical domains was found to be highly variable (Fig. 1B). These observations indicate that Keap1 has some flexible regions, resulting in multiple conformations in the native state. These problems resulted in a poor 3D reconstruction and a subsequent reduction in resolution in the single-particle analysis of Keap1.

Most of the observed circular images had doughnut-like shapes, with a central low-density region (Fig. 1B). The diameter of each doughnut image is approximately 50 Å. This observation indicates that these circular images are derived from the DC domain, which consists of the six-bladed β-propeller structure (Fig. 1C, D) [15,16,24,25,32]. The overall size and shape of the DC domain densities in this cryo-EM study are highly consistent with our previously determined crystal structures. Moreover, these data suggest that the DC domain of Keap1 exhibited highly preferred orientation.

### 2.2 A novel method for analyzing the inter-domain distance of two DC domains

We hypothesized that the variability in the distance between the DC domains, as observed in the 2D class averages, could serve as a measure for inferring the structural changes of Keap1. To this end, we developed a novel method for analyzing the inter-domain distance of two DC domains by fitting atomic models of the DC domain onto 2D class-average images (Fig. 1E).

As shown in the top panels of Fig. 1E Step 1, we created low-resolution projections of a single DC domain in various orientations by rotating the atomic model of the DC domain (PDB ID: 3vnh, chain A). Using these projections as templates, we then matched them to 2D class-average images from cryo-EM particle images. As shown in the bottom panels of Fig. 1E Step 2, we obtained the information on the positions and orientations of the DC domains for each image. Finally, we estimated the center-to-center distance between the two DC domains from the matched DC domains using particle population-weighted statistics. No estimations were performed when three domains were observed.

### 2.3 The inter-domain distance analysis enabled quantitative assessment of structural changes in Keap1 dimer upon Neh2 binding

**Fig. 2:**
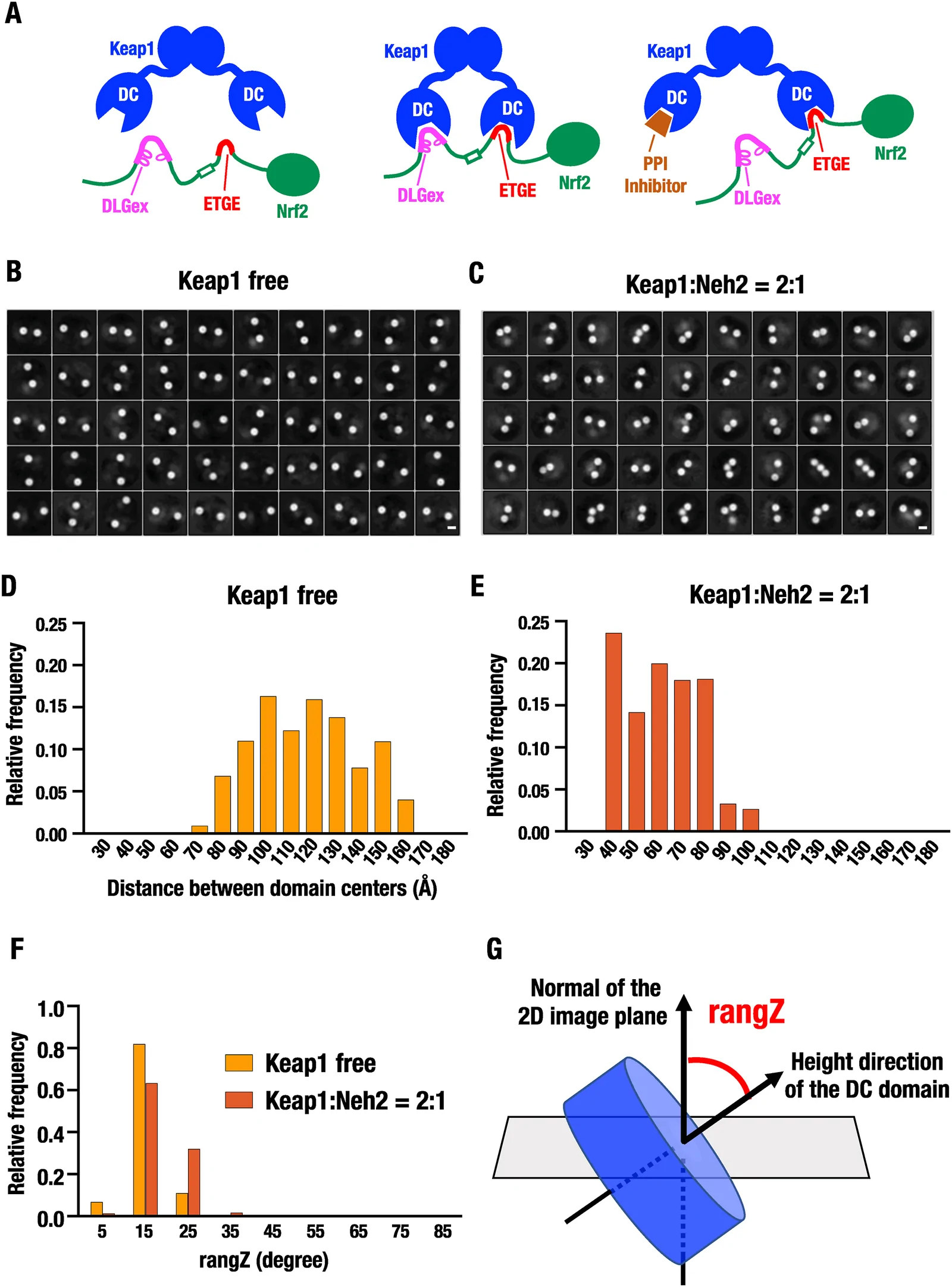
**Analysis of the distance between two DC domains in Keap1 alone form and in Keap1-Neh2 mixture.** (A) Proposed binding configurations of the Keap1-Nrf2 complex. Based on the substantially higher affinity of the ETGE motif for the Keap1 DC domain compared with the DLGex motif, three representative interaction states are illustrated: the unbound state (left), the fully engaged state with both motifs bound (middle), and the ETGE-only bound state where a PPI inhibitor (square) takes the place of the DLGex motif in the binding site of the DC domain (right). (B) and (C) Representative 2D class averages of Keap1 alone form (B) and of the Keap1-Neh2 mixture (2:1 molar ratio: C). Scale bar (bottom right): 50 Å. (D) and (E): Distributions of inter-DC domain distance for Keap1 alone form (D) and the Keap1-Neh2 mixture (2:1 molar ratio: E). The X-axis represents the distance between the two DC domains (Å), and the Y-axis represents the relative frequency. (F) Distributions of the angle (rangZ) between the axial height direction of the DC domain and the normal vector of the 2D image plane. The X-axis represents rangZ (degree), and the Y-axis represents the relative frequency. The histograms are derived from the same samples shown in panels D and E. (G) Schematic explanation of rangZ.

To investigate the structural change of the Keap1 homodimer upon binding of the Nrf2 Neh2 domain, we applied the novel inter-domain distance analysis system to the 2D class averages of both Keap1 homodimer alone and a 2:1 mixture of Keap1 homodimer and Neh2 domain. The Neh2 domain contains two Keap1-binding motifs, DLGex and ETGE [30], which bind to each DC domain of the Keap1 homodimer individually (Fig. 2A). Therefore, the distance between the two DC domains of Keap1 homodimer must be changed in the presence of the Neh2 domain. To test this hypothesis and to clarify the change in the distance between two DC domains upon binding of the Neh2 domain, we compared 2D class averages of Keap1 alone and a mixture of Keap1 and Neh2. Representative micrographs and 2D class averages and the data processing workflow are shown in Supplementary Fig. S1, and the number of particles at each step is summarized in Supplementary Table S1. Purified full-length Keap1 and Neh2 proteins used for the following cryo-EM analyses are shown in Supplementary Fig. S2. Size-exclusion chromatography showed a single, symmetric peak corresponding to the Keap1 homodimer, indicating that the purified Keap1 exists as a stable homodimer in solution.

The cryo-EM analysis revealed that Keap1 alone displayed the particles with highly variable and relatively open inter-domain distances in the 2D class average (Fig. 2B). By contrast, the 2:1 mixture of Keap1 and Neh2 showed particles with a closer inter-domain distance compared to Keap1 alone (Fig. 2C). The distances between two DC domains without Neh2 were broadly distributed between 70 Å and 160 Å (Fig. 2D), while those in the 2:1 Keap1:Neh2 mixture were shifted toward a relatively narrower range of 40 to 100 Å (Fig. 2E). Thus, our novel approach indeed supports the quantitative characterization of conformational changes in the Keap1 homodimer. While it has been shown that two Keap1 DC domains and ETGE and DLGex motifs in one Neh2 domain of Nrf2 form a two-site binding complex [13,15,16,18,30], these results for the first time demonstrate that binding of the Neh2 domain to the Keap1 homodimer makes the distance between two DC domains narrow. The Neh2 domain itself was not resolved in the 2D class averages, likely due to the limited resolution in the present analysis, as well as its small size and the flexibility of the linker between the DLGex and ETGE motifs.

In addition, we analyzed the angles (rangZ) between the height direction of the DC domain and the normal direction of the 2D image plane. Because the DC domain appears short cylinder-like at low resolution, its orientation can be characterized solely by the axial height direction of the cylinder (Z-axis). Through the analysis, we found that those angles were strictly limited in between 15° and 25°, indicating that the DC domains have a strong preference for face-up orientations (Fig. 2F, G).

### 2.4 Inter-domain distance analysis of Keap1-Neh2 mixture with a protein-protein interaction inhibitor

We further applied this inter-domain distance analysis system for the evaluation of the effect of a new Nrf2 inducer (*i.e.,* Keap1 inhibitor), CH7450924, on the Keap1-Neh2 complex structure [33]. CH7450924 is supposed to induce Nrf2 stability via inhibition of the protein-protein interaction (PPI) between Keap1 and Neh2 domain, thereby reducing the ubiquitin ligase activity of Keap1. To experimentally validate this notion, we added CH7450924 to the Keap1-Neh2 domain complex and conducted cryo-EM analysis.

**Fig. 3:**
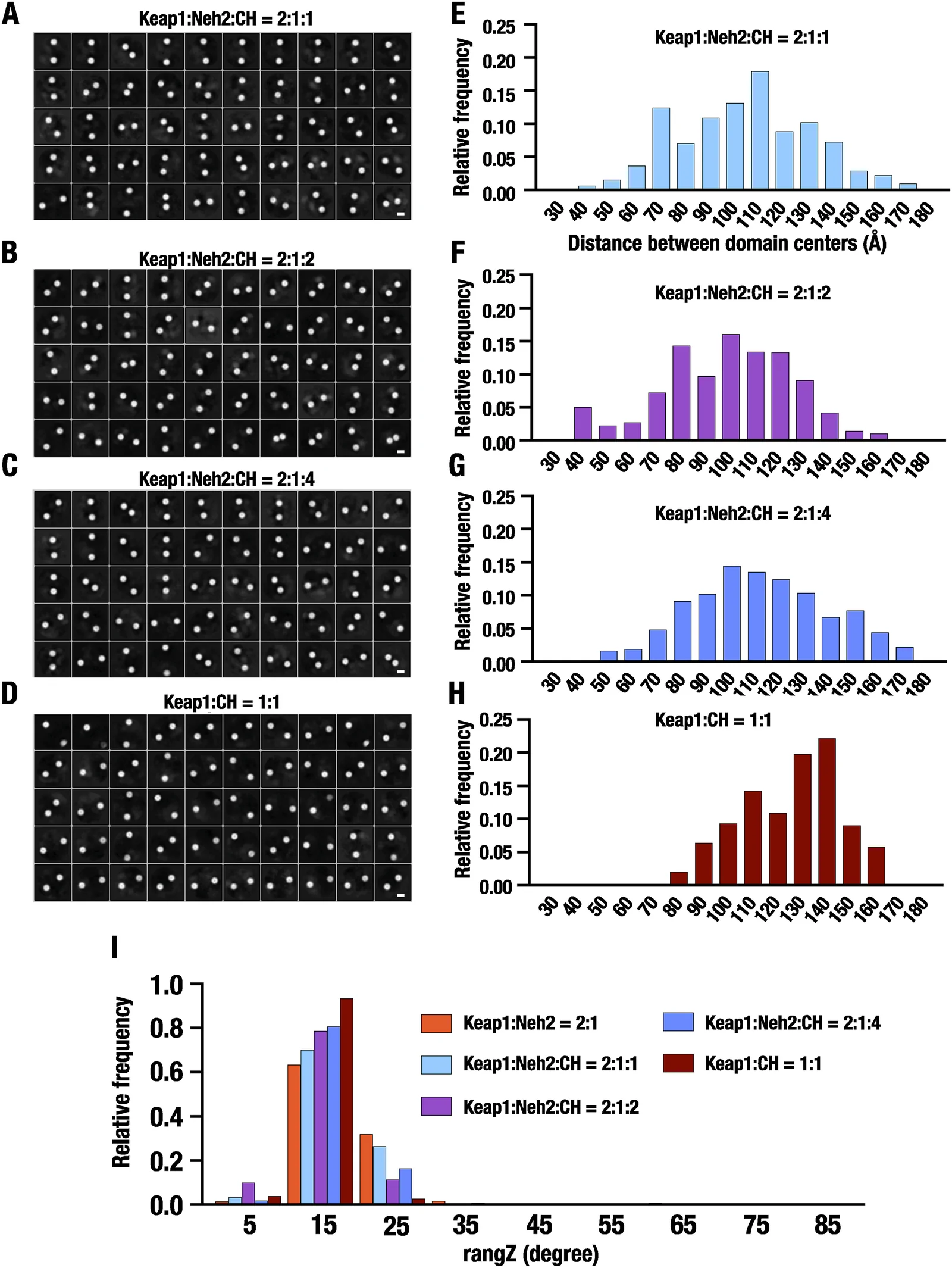
**Analysis of the distance between two DC domains in the Keap1-Neh2-CH7450924 mixtures.** (A–D) Representative 2D class averages of the Keap1 samples with Neh2 and/or the protein-protein interaction inhibitor CH7450924 (CH). Scale bar (bottom right of each panel): 50 Å. (E–H) Distributions of inter-DC domain distance for the corresponding Keap1 samples. The X-axis represents the distance between the two DC domains (Å), and the Y-axis represents the relative frequency. The molar ratios were as follows: (A) and (E), Keap1:Neh2:CH = 2:1:1; (B) and (F), Keap1:Neh2:CH = 2:1:2; (C) and (G), Keap1:Neh2:CH = 2:1:4; and (D) and (H), Keap1:CH = 1:1. (I) Distributions of the angle (rangZ) between the axial height direction of the DC domain and the normal vector of the 2D image plane. The X-axis represents rangZ (degree), and the Y-axis represents the relative frequency. The histograms are derived from the samples shown in panels (A–D) and Fig. 2C.

The addition of CH7450924 to the Keap1-Neh2 mixture at half the concentration of Keap1 (*i.e.,* Keap1:Neh2:CH7450924 = 2:1:1) dramatically increased the population of particles harboring an enlarged inter-DC domain distance (Fig. 3A, E). Of note, this distribution of inter-DC domain distance was much wider than that of Keap1-Neh2 complex (Fig. 2C, E), but moderately closer to that of Keap1 alone (Fig. 2B, D).

It has been shown that DLGex motif retains approximately 100-fold weaker affinity with the Keap1 DC domain than that of ETGE motif, which makes the molecular basis of the interaction of Keap1 homodimer and Neh2 domain or the Hinge and Latch model [30]. The present result demonstrates that the binding of two sites (*i.e.,* the Hinge and Latch) in the Neh2 domain tightens the two DC domain distance in the Keap1 homodimer, but the disruption of two-site binding at one site (*i.e.,* DLGex motif or the Latch site) brings the enlargement of the inter-DC domain distance, through disrupting the two-site binding of Keap1 DC domains and Neh2 domain (Fig. 2A, right panel).

To further characterize how the disruption of the two-site binding correlates with the enlargement of inter-DC domain distance, we next examined the addition of CH7450924 to the Keap1-Neh2 mixture at an equimolar concentration of Keap1 (Keap1:Neh2:CH7450924 = 2:1:2). This condition generates CH7450924 binding to both DC domains in the Keap1 homodimer. The addition resulted in an inter-DC domain distance distribution of 40 to 160 Å, which was peaked at 80 to 130 Å (Fig. 3B, F), which is the similar result to the addition of CH7450924 at half the concentration of Keap1 (Fig. 3A, E). This result clearly demonstrates that two-site disruption causes similar enlargement of the inter-DC distance to one-site disruption of Keap1 DC domain and Neh2 domain. This result supports the Hinge-Latch mechanism in which dissociation of one site (the Latch site) is sufficient to provoke inactivation of the Keap1 ubiquitin ligase activity to Nrf2 [18,30].

The addition of CH7450924 at a 2-fold higher concentration than the Keap1 homodimer (Keap1:Neh2:CH7450924 = 2:1:4) led to a slight increase in the proportion of the particles with widely separated DC domains, while decreasing those with the shorter inter-DC domain distance (Fig. 3C, G). However, these changes are moderate and the efficacy of CH7450924 is saturated with two-fold excess addition, indicating strong binding affinity of CH7450924 to the DC domain of Keap1.

The addition of an equal amount of CH7450924 to Keap1 alone did not significantly affect the distribution of the distance between the two DC domains, compared to that of Keap1 alone (Fig. 3D, H). In addition, the rangZ values were still strictly limited in between 15° and 25° for all CH7450924 titration experiments (Fig. 3I), supporting the validity of our approach in the CH7450924 titration experiments. These results demonstrate that CH7450924 inhibits the interaction between Keap1 DC domain and Nrf2 Neh2 domain or the Hinge and Latch structure.

### 2.5 Keap1 preferentially distributes at air-water interface and PEGylation mitigates the preferred distribution

To investigate the underlying cause of the preferred orientation of Keap1, we performed cryo-electron tomography (cryo-ET) to verify whether Keap1 was preferentially localized at the Air-Water Interface (AWI). Cryo-ET has been used to reconstruct 3D tomographic data from a tilt series of images collected at a range of continuous tilts, resulting in tomographic data allows for the three-dimensional localization of particles [34].

**Fig. 4:**
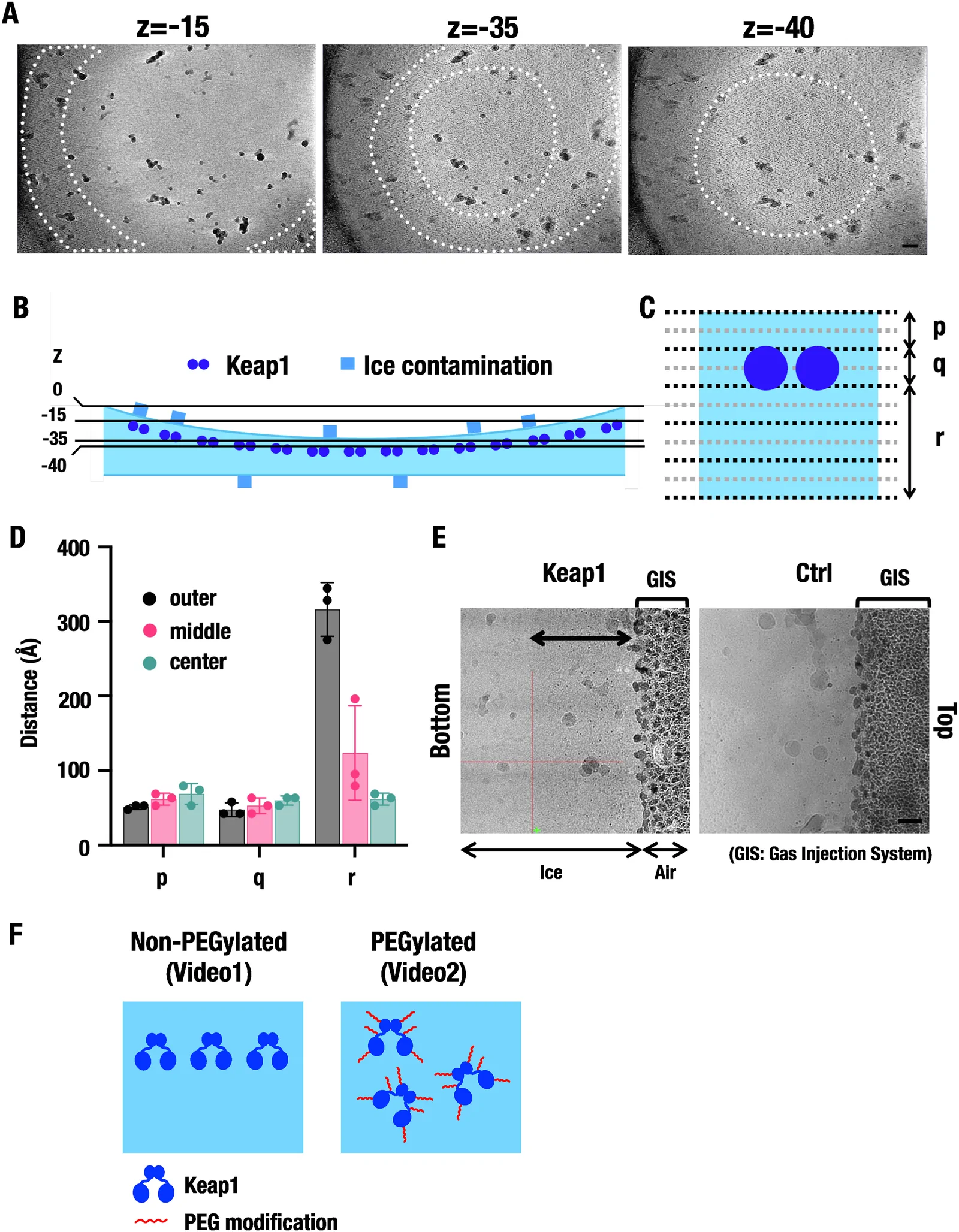
**Preferential localization of Keap1 homodimers at the air-water interface in amorphous ice.** (A) Tomography data of full-length Keap1 in vitreous ice suspended over a grid hole. Keap1 particles are observed within the region delineated by the dashed line. Images focused at Z depths of −15 (left panel), −35 (center panel), and −40 (right panel), respectively. Scale bar (bottom right): 50 nm. (B) Schematic representation of Keap1 particles within vitreous ice spanning a grid hole. The blue spheres illustrate the positions of the Keap1 DC domains. The region of the vitreous ice, including the ice contamination, is colored cyan. The Z-value indicates the slice number in the tomogram, where a value of 0 is defined as the ice surface closest to the top of the grid. (C) Schematic diagram of the distribution of Keap1 DC domains within the ice. Label p: The distance from the top ice surface to the top of the DC domain distribution. Label q: Vertical range of the DC domain distribution. Label r: The distance from the bottom of the DC domain distribution to the bottom ice surface. The blue spheres illustrate the positions of the Keap1 DC domains. (D) Distributions of the Keap1 DC domain position in the vitreous ice. The labels p, q, and r correspond to the parameters defined in Fig. 4C. The classification of “outer”, “middle”, and “center” refers to distinct regions within the EM grid hole. These three regions were defined by the approximate distance from the center of the hole (d): “center” (0 < d < 1/3R), “middle” (1/3R < d < 2/3R), and “outer” (2/3R < d < R), where R represents the radius of the hole. All distances were calculated based on the reconstructed tomogram thickness and the pixel size. (E) Cryo-TEM image of a thin lamella prepared by cryo-FIB milling at 30,000× magnification. The black arrow indicates the range of Keap1 particle distribution. Scale bar (bottom right): 50 nm. The gas injection system (GIS) was used to deposit a protective platinum layer. (F) Schematic diagram of the spatial distribution of Keap1 molecules in vitreous ice for non-PEGylated (left) and PEGylated (right) samples. The blue shapes illustrate the positions of the Keap1 molecules, with the PEG modifications indicated by red wavy lines.

As we moved the z-slice position of the reconstructed 3D tomographic data from the top to the bottom of the grid, the distribution of particle images considered to be Keap1 particles shifted from the outer to the inner part of the field of view (Fig. 4A, Video 1). This observation indicates both *i*) the concave shape of the ice surface toward the center of the grid and *ii*) the preferential distribution of Keap1 particles at the AWI as illustrated in the schematic diagram (Fig. 4B).

Supplementary data related to this article can be found online at https://doi.org/10.1016/j.redox.2026.104379

The following are the Supplementary data related to this article:

Video 1 **Reconstructed 3D tomographic data of Keap1 visualized by Z-axis slicing.**
This video shows successive slices through 3D reconstructed data of Keap1 in ice, with the slices passing sequentially through the volume along the Z axis. The distribution of particle images of Keap1 particles shifted from the outer to the inner part of the field of view. Large, heterogeneous particles are ice contaminations. Scale bar: 50 nm.

We also analyzed tomographic data to estimate the distance from the surface of the ice to the top of DC domain distribution (*p*), the range of the DC domain distribution (*q*), and the distance from the DC domains to the bottom ice surface (*r*) (Fig. 4C). As a result, regardless of the position within the hole, the distance *p* was relatively short and constant, ranging from 51 Å to 69 Å, whereas the distance *r* was diverse, ranging from 62 Å to 316 Å (Fig. 4D). The DC domain distribution range, *q*, was consistent across all positions, similar to that observed in *p*, indicating that Keap1 particles are preferentially distributed at the AWI on the top side of the ice. The distance *r* is considered to reflect the ice thickness, which is likely the cause of the variability since *r* becomes larger in thicker ice.

To more extensively investigate the behavior of Keap1 in ice, a thin lamella was prepared from a frozen Keap1 solution on a grid using a cryo-FIB-SEM, and was then observed by TEM. The TEM images revealed an accumulation of particles at the AWI of the lamella (Fig. 4E). The diameters of these particles indicate that these particles are derived from Keap1. These data show the preferential distribution of Keap1 particles at the AWI.

Chemical modification of some proteins with polyethylene glycol (PEG) is known to improve particle dispersion and mitigate preferred orientation in cryo-EM samples [35]. Therefore, to investigate the PEGylation effect of Keap1 on its orientation in vitrified ice, we conducted tomographic imaging of the PEGylated Keap1 sample. We found that Keap1 particles were distributed uniformly throughout the ice layer, from the top to the bottom surfaces (Fig. 4F; Video 2). This observation shows that PEGylation of Keap1 attenuated the particle adsorption to AWI, compared to the non-PEGylated Keap1.

Supplementary data related to this article can be found online at https://doi.org/10.1016/j.redox.2026.104379

The following are the Supplementary data related to this article:

Video 2 **Reconstructed 3D tomographic data of PEGylated Keap1 visualized by Z-axis slicing.**
This video shows successive slices through 3D reconstructed data of PEGylated Keap1 in ice, with the slices passing sequentially through the volume along the Z axis. Note that the particles of PEGylated Keap1 were distributed uniformly throughout the ice layer, from the top to the bottom surfaces. Scale bar: 50 nm.

### 2.6 Inter-domain distance analysis of PEGylated Keap1 samples

**Fig. 5:**
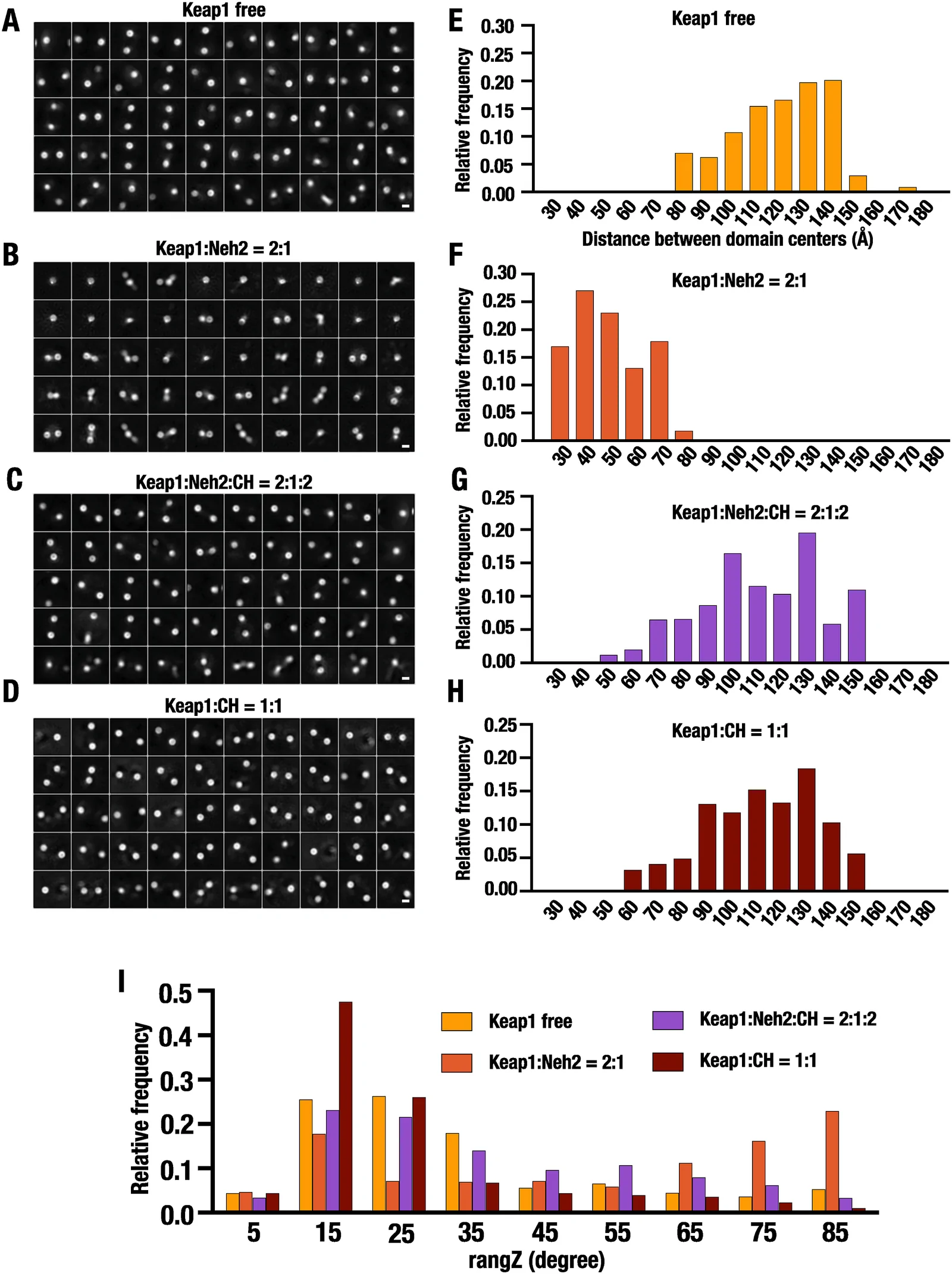
**Analysis of the distance between DC domains in the PEGylated Keap1-Neh2-CH7450924 mixture.** (A–D) Representative 2D class averages of the PEGylated Keap1 free form (A), the PEGylated Keap1:Neh2 mixture (B), the PEGylated mixture of Keap1:Neh2:CH7450924 (CH) (C), and the PEGylated Keap1:CH mixture (D). Scale bar (bottom right of each panel): 50 Å. (E–H) Distributions of inter-DC domain distance for the corresponding PEGylated Keap1 samples. The X-axis represents the distance between the two DC domains (Å), and the Y-axis represents the relative frequency. The molar ratios were as follows: (A) and (E), PEGylated Keap1 free form; (B) and (F), PEGylated Keap1:PEGylated Neh2 = 2:1; (C) and (G), PEGylated Keap1:PEGylated Neh2:CH = 2:1:2; and (D) and (H), PEGylated Keap1:CH = 1:1. (I) Distributions of the angle (rangZ) between the axial height direction of the DC domain and the normal vector of the 2D image plane. The X-axis represents rangZ (degree), and the Y-axis represents the relative frequency. The histograms are derived from the same samples shown in panels (A–D).

To assess reproducibility of our inter-domain distance analysis, we conducted an inter-DC domain distance analysis utilizing the PEGylated Keap1. We found that the distances between two DC domains were distributed between 80 Å and 140 Å (Fig. 5A, E), which is similar to those of the non-PEGylated form (see Fig. 2B, D). Thus, the PEGylated Keap1 alone reproducibly exhibited an open conformation between two DC domains in the 2D class average images.

In the analysis of inter-DC domain distances for the PEGylated Keap1-Neh2 complex (Keap1:Neh2 = 2:1), we found diverse morphologies including several new 2D image classes, such as those containing particles exhibiting non-globular shapes, in addition to 2D image class containing only DC domains (Fig. 5B). The 2D images exhibiting non-globular shapes seem to indicate that the other domains of Keap1 (*i.e.,* the BTB and IVR domains) may also be observed in the case of the PEGylated Keap1-Neh2 complex. For the subsequent inter-DC domain distance analysis, only 2D image classes containing two clearly visible DC domains were included, whereas classes containing one or three visible DC domains were excluded from further analysis. Details of the particle classification analysis of the PEGylated samples, including the rationale for excluding classes containing one or three visible DC domains and the proportions of each class category, are available in the Supplementary information (Supplementary note 1, Supplementary Table S4, and Supplementary Fig. S3).

To our expectation, this 2D image analysis again revealed that the inter-domain distance of the PEGylated Keap1-Neh2 complex (Keap1:Neh2 = 2:1) was shorter than that of PEGylated Keap1 alone. The inter-domain distances of the PEGylated Keap1-Neh2 complex were distributed between 30 and 70 Å (Fig. 5F), much shorter than those for PEGylated Keap1 alone (Fig. 5E), but similar to those for the non-PEGylated Keap1-Neh2 complex (see Fig. 2E). These results demonstrate that the inter-DC domain distance of the Keap1 decreases upon interaction with Neh2, even if the Keap1 was PEGylated.

We further analyzed the effect of CH7450924 on changes in the inter-DC domain distances of the PEGylated Keap1-Neh2 complex. In the case of the PEGylated condition of the 2:1:2 mixture (Keap1:Neh2:CH7450924 = 2:1:2), the distances were widened again, which were distributed between 50 and 150 Å (Fig. 5C, G). This distribution was very similar to that of non-PEGylated sample (see Fig. 3B, F), indicating that, even in the condition of Keap1 and Neh2 PEGylation, the addition of CH7450924 exhibited the power to inhibit interaction between Keap1 and Nrf2, and the inter-DC domain distance of Keap1 returned to similar distance to that of Keap1 free form. Addition of CH7450924 to the PEGylated Keap1 alone did not change substantially the distribution of the inter-DC domain distance (Fig. 5D, H).

Taken together, these results indicate that the changes in the distribution of the inter-DC domain distance of Keap1 caused by the addition of the Neh2 domain and CH7450924 were not affected substantially by the PEGylation.

It should be noted that when we investigated the rangZ of the PEGylated samples, the angles between the height direction of the DC domain and the normal direction of the 2D image plane for these PEGylated samples (Fig. 5I) showed marked changes compared with those of non-PEGylated samples (Figs. 2F and 3I). The distributions of rangZ values of the PEGylated samples exhibited broad and characteristic profiles (Fig. 5I), showing that PEGylation also improved the orientational diversity of the DC domains.

### 2.7 Keap1 contains multiple regions that elicit conformational flexibility

**Fig. 6:**
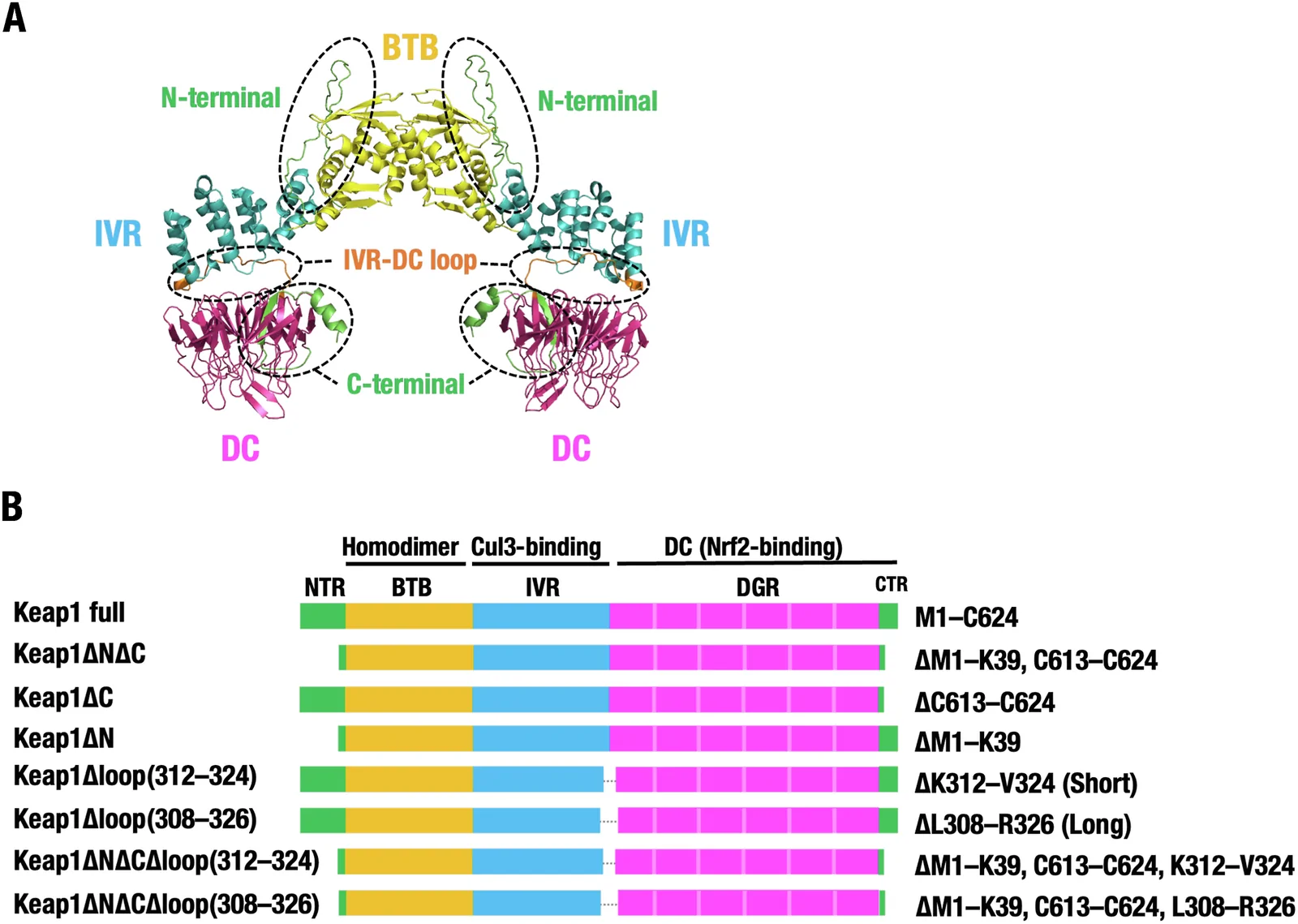
**Predicted structural model of Keap1 and schematic representation of deletion mutants.** (A) Predicted structural model of the Keap1 homodimer by AlphaFold2 [36]. The BTB (broad complex, tramtrack and bric-a-brac) domain is colored yellow, the Intervening region (IVR) cyan, and the DC (Kelch plus C-terminal) domain magenta. The N-terminal region (NTR) and the C-terminal region (CTR) are shown in green. (B) Schematic representation of Keap1 and its deletion mutants. Each domain is colored as described in Panel (A). The deleted regions, including NTR, CTR, and the IVR-DC domain loop, are indicated.

In the next step, we aim to reduce the conformational heterogeneity in order to determine the high-resolution structure of Keap1. To this end, we investigated multiple regions within Keap1 that may contribute to eliciting structural changes of Keap1. As shown in Fig. 6A, we first predicted three-dimensional structure of full-length Keap1 using the AlphaFold2 program. The predicted structure and our previous analyses [27] suggest that there are three flexible regions in Keap1: the N-terminal region (residue number: 1–39), the C-terminal region (residue number: 613–624), and the loop region between the IVR and the DC domains (residue number: 308–326). We hypothesized that these three flexible regions bring about the structural diversity of Keap1. Therefore, we prepared seven deletion mutants of Keap1 (Fig. 6B) and analyzed them by the present inter-domain analysis system.

### 2.8 Deletions of the N-terminal and/or C-terminal regions reduce inter-DC domain distance

To test our hypothesis on these flexible regions, we investigated how deletions of N-terminal and/or C-terminal regions of Keap1 affect the inter-DC domain distance. SDS-PAGE analyses of the purified deletion mutants used in this study are shown in Supplementary Fig. S4. To our expectation, we found that all three deletion mutants, *i.e.,* deletions of the N-terminal region (Keap1ΔN), C-terminal region (Keap1ΔC), and both regions (Keap1ΔNΔC), showed the particles with a shorter inter-DC domain distance, compared to those of full length Keap1.

**Fig. 7:**
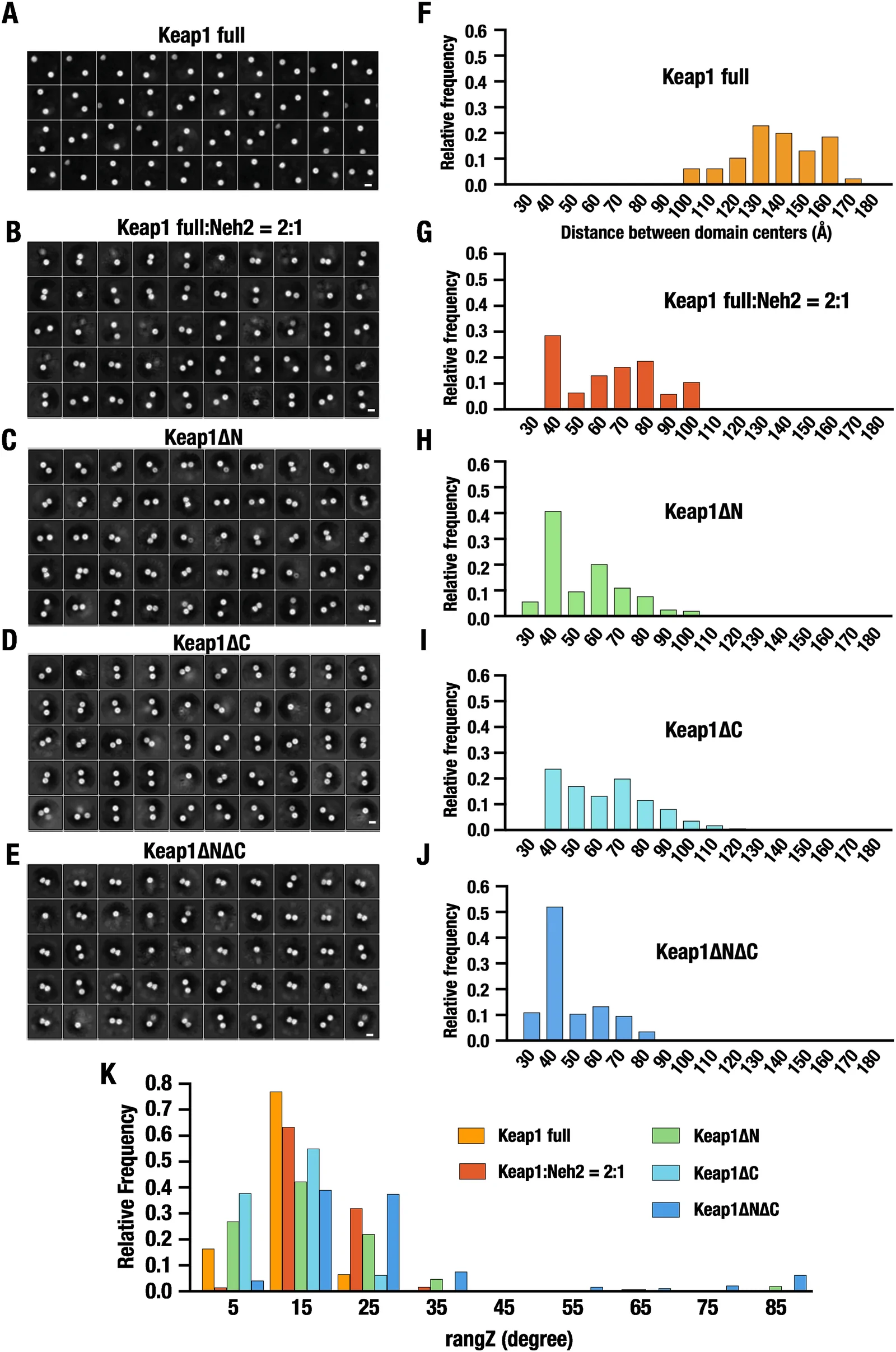
**Effects of N- and C-terminal deletions on the inter-DC domain distance and molecular orientation of the Keap1 homodimer.** (A–E) Representative 2D class averages of the full-length Keap1 (A), the Keap1-Neh2 mixture (2:1 molar ratio: B), the Keap1 N-terminal deletion mutant (Keap1ΔN, C), the Keap1 C-terminal deletion mutant (Keap1ΔC, D), and the Keap1 mutant with both N- and C-terminal deletions (Keap1ΔNΔC, E). Scale bar (bottom right of each panel): 50 Å. (F–J) Distributions of inter-DC domain distance for the corresponding Keap1 samples. The X-axis represents the distance between the two DC domains (Å) and the Y-axis represents the relative frequency. (K) Distributions of the angle (rangZ) between the axial height direction of the DC domain and the normal vector of the 2D image plane. The X-axis represents rangZ (degree), and the Y-axis represents the relative frequency. The histograms are derived from the same samples shown in panels (A–E).

Displaying a very good reproducibility to the experiments in the former section (see Fig. 2), the inter-DC domain distance of full-length Keap1 exhibited wide distance, mean value of which was 140 Å (Fig. 7A, F). Addition of Neh2 domain reduced the distance to mean value of 70 Å (Fig. 7B, G). Of note, deletion of Keap1 N-terminal (Keap1ΔN) or C-terminal (Keap1ΔC) region resulted in marked reductions of the inter-domain distance (Fig. 7C, H and D, I, respectively) compared with that of full-length Keap1 (Fig. 7A, F). Intriguingly, the reduction was more significant than that provoked by the addition of Neh2 domain (Fig. 7B, G).

A salient observation here was that both Keap1 N-terminal and C-terminal deletions (Keap1ΔNΔC) led to an increase in the proportion of the particles with an inter-DC domain distance of approximately 40 Å (Fig. 7E, J), which was much shorter than the cases of Keap1ΔN or Keap1ΔC. In addition, Keap1ΔNΔC reduced range of the distribution of the inter-domain distance. These results support our contention that both the N-terminal and C-terminal regions influence the flexibility of the Keap1 structure, while concomitant deletion of both regions influences the flexibility much larger than those by the N-terminal and C-terminal regions independently.

In addition, the distribution of rangZ value for Keap1 deletion mutants was somewhat broader than that for full length Keap1, while rangZ values of most particles still fall within the range of 5° to 25° (Fig. 7K). These results suggest that the deletions of the N-terminal and the C-terminal regions give rise to limited influence on the orientation of Keap1.

### 2.9 Deletion of the loop region between IVR and DC domains also reduces the inter-domain distance

Given the fact that deletion of Keap1 N-terminal or C-terminal region resulted in marked reductions of the inter-domain distance, we hypothesized that deletion of the loop region between IVR and DC domains of Keap1 may also affect the inter-DC domain distance. To address how deletion of the loop region affects the inter-DC domain distance, we constructed two types of deletions of the IVR-DC loop (see Fig. 6B). One was the short deletion of Δloop(312–324) and the other was the long deletion of Δloop(308–326). SDS-PAGE analyses of the purified loop deletion mutants used in this study are shown in Supplementary Fig. S5.

**Fig. 8:**
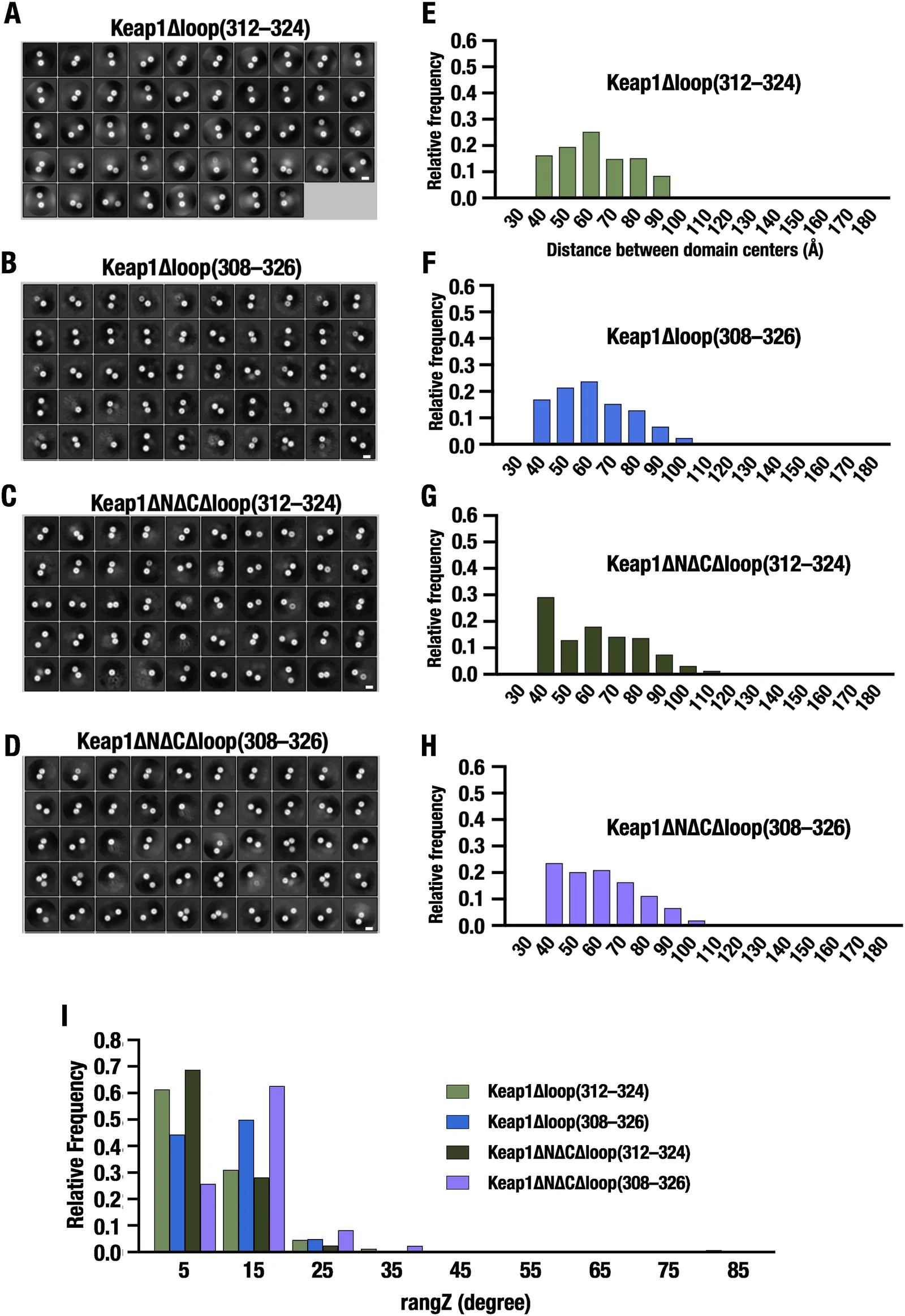
**Effect of loop deletions on the inter-DC domain distance and molecular orientation of the Keap1 homodimer.** (A–D) Representative 2D class averages of the Keap1 mutants deleting the loop region between IVR and DC domains, in combination without (A, B) or with (C, D) both N- and C-terminal deletions. The mutants are as follows: (A) short loop deletion (Keap1Δloop(312–324)), (B) long loop deletion (Keap1Δloop(308–326)), (C) short loop deletion combined with N- and C-terminal deletions (Keap1ΔNΔCΔloop(312–324)), and (D) long loop deletion combined with N- and C-terminal deletions (Keap1ΔNΔCΔloop(308–326)). (E–H) Distributions of inter-DC domain distance for the corresponding Keap1 samples. The X-axis represents the distance between the two DC domains (Å), and the Y-axis represents the relative frequency. (I) Distributions of the angle (rangZ) between the axial height direction of the DC domain and the normal vector of the 2D image plane. The X-axis represents rangZ (degree), and the Y-axis represents the relative frequency. The histograms are derived from the same samples shown in panels (A–D).

To our surprise, both deletions reproducibly shortened the inter-DC domain distance to the mean value of 62 Å (Fig. 8A, E and B, F, respectively). No significant difference of the distribution was found between the short and long deletions of the IVR-DC loop. The mean values of the inter-DC domain distance of these loop deletions were similar to the Keap1-Neh2 mixture but moderately longer than the N-terminal (Keap1ΔN) and C-terminal (Keap1ΔC) deletions.

We finally investigated the effect of all three-sites deletions, *i.e.,* the N-terminal, C-terminal, and IVR-DC loop regions, on the inter-DC domain distance (Fig. 8C, G and D, H, respectively). The all three-sites deletions also resulted in a shorter inter-DC domain distance, the mean value of 61 Å and 60 Å for the shorter deletion and longer deletion, respectively. These values were basically similar to those of the IVR-DC loop deletions. In addition, the distributions of rangZ for all loop deletions did not change significantly from that of full length Keap1, limited in between 5° and 25° (Fig. 8I). Taken together, these results indicate that the N-terminal, C-terminal, and IVR-DC loop regions all influence the flexibility of the DC domains.

## 3 Discussion

**Fig. 9:**
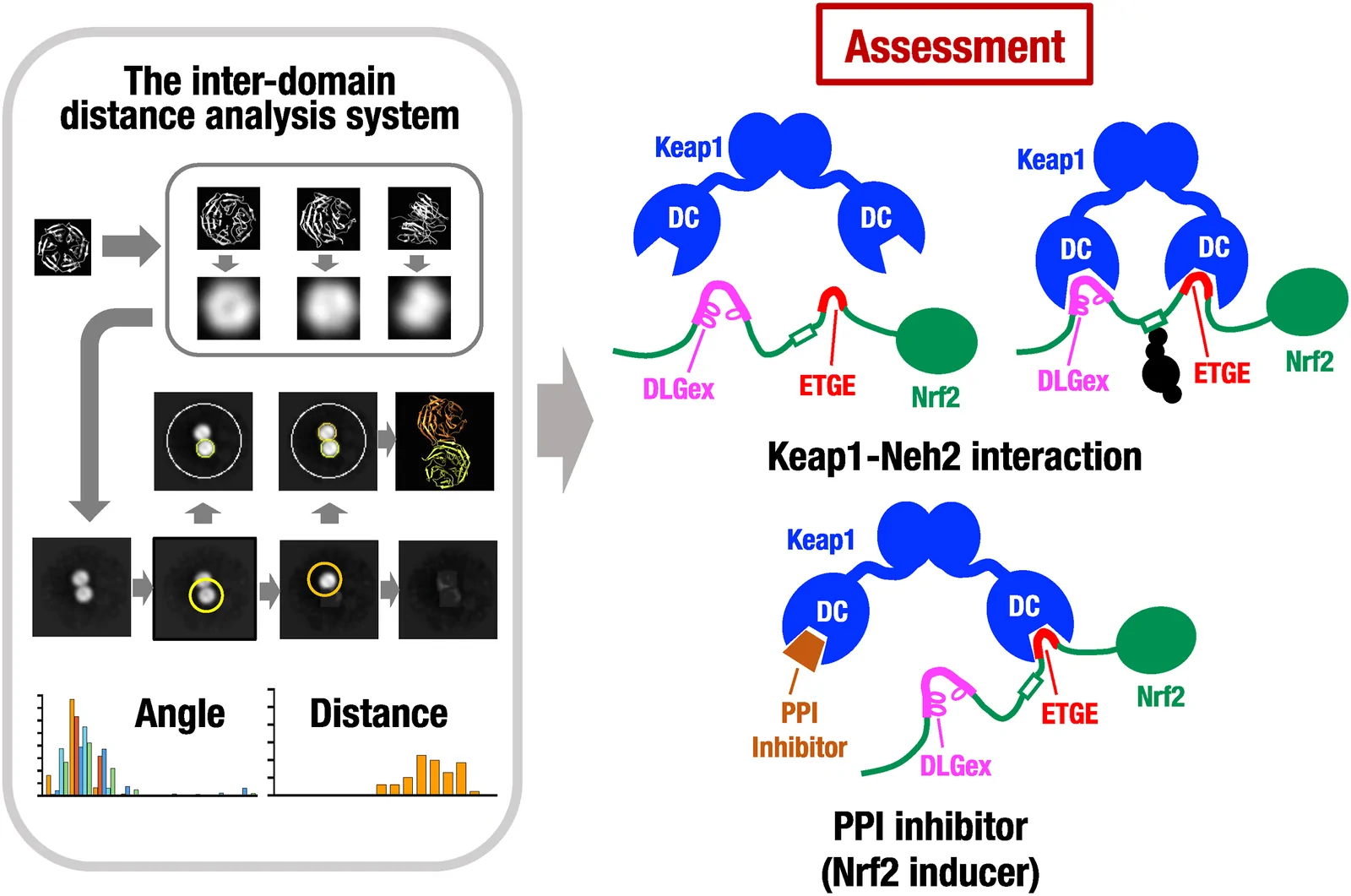
Overview of the conformational changes in the Keap1-Nrf2 system suggested by our new domain distance analysis system based on cryo-EM data.

In this study, we established an inter-domain distance analysis system that quantitatively evaluates structural changes of the Keap1 homodimer in response to binding of the Nrf2 Neh2 domain and a PPI inhibitor. As shown in Fig. 9, our results demonstrate that simultaneous engagement of the two DC domains of the Keap1 homodimer to the DLGex and ETGE motifs in the Nrf2 Neh2 domain reduces the inter-domain distance between the DC domains. Titration experiments combined with this analytical system further revealed that a newly synthesized PPI inhibitor of the Keap1-Nrf2 complex, CH7450924, disrupts one or both interactions between the Keap1 DC domain and Nrf2 Neh2 domain, thereby increasing the distance between two DC domains. To overcome the preferred orientation in the vitrified ice, we employed PEG, which enabled reproducible measurements. In addition, we found that three regions in Keap1, *i.e.,* the N-terminal, C-terminal, and IVR-DC loop regions, which were predicted to be unstructured in AlphaFold2 program, significantly influence DC domain flexibility. Collectively, these findings indicate that binding of the Nrf2 Neh2 domain induces structural rearrangements within the Keap1 homodimer, whereas these changes are abolished upon inhibition of the DC-Neh2 interaction by the PPI inhibitor. Thus, structural rearrangements appear to be a critical mechanistic feature underlying Keap1 function. This study represents an important advance in our understanding of the Keap1-Nrf2 interaction mechanism and provides a solid foundation for future high-resolution structural analysis of Keap1.

The salient feature of this study is that our new cryo-EM-based analysis system quantifies the changes in the inter-domain distance within the Keap1 dimer, which provides insights into overall structural rearrangements of Keap1. In our previous NMR study, we monitored changes in the NMR signals of the Nrf2 Neh2 domain upon addition of full-length Keap1 and/or PPI inhibitors [18]. That study demonstrated that the two binding motifs within the Neh2 domain interact independently with full-length Keap1, with distinct binding affinities, supporting the previously proposed Hinge-Latch mechanism [15,16,18].

In contrast, the present study focuses on conformational changes in full-length Keap1 induced by Nrf2 binding, achieved through direct visualization of the DC domains of Keap1 by cryo-EM. While the conformational heterogeneity and severe preferred orientation hindered the reconstruction of the 3D overall potential map of Keap1, our results demonstrate, for the first time, that Keap1 dimer undergoes a ligand-induced change in inter-DC domain distance upon Nrf2 binding, thereby providing novel and complementary structural insight into the Keap1-Nrf2 interaction. These findings support the Hinge-Latch mechanism as the molecular basis for the inactivation of Keap1 ubiquitin ligase activity and consequent activation of Nrf2.

The inter-DC domain distance analysis system developed in this study capitalizes on the pronounced preferred orientation of Keap1 particles in cryo-EM. Cryo-ET analysis revealed that most Keap1 molecules are distributed at depths from 50 Å to 100 Å below the ice surface on the EM grid, suggesting adsorption to the air-water interface. Although the precise mechanism underlying this behavior remains unclear, Keap1 particles exhibit a highly preferred orientation at this interface. Consistent with our observations, previous cryo-ET studies have reported that approximately 90% of particles in vitreous ice localize at depths from 50 to 100 Å of the ice surface [37]. Furthermore, cryo-FIB-SEM analysis independently confirmed the preferential localization of Keap1 at the AWI. Recent advances in cryo-FIB-SEM technology have enabled reproducible preparation of lamellae even from vitrified protein samples [38]. Leveraging this capability, we directly examined the spatial distribution of protein particles embedded in vitreous ice. These findings underscore the utility of cryo-FIB-SEM for characterizing particle distribution and highlight its broader applicability in structural studies of proteins prone to interfacial adsorption.

Our analysis revealed a broad distribution of inter-DC domain distances in Keap1 alone, spanning approximately 70–160 Å. In contrast, a previous study using negative staining EM reported a DC-DC distance of approximately 80 Å for mouse KEAP1 [27]. This discrepancy likely reflects methodological differences, including sample preparation and the number of particles analyzed. Negative staining EM requires heavy metal staining and dehydration, procedures that can potentially constrain conformational flexibility. Moreover, the earlier three-dimensional reconstruction was based on approximately 10,000 particles. In contrast, our cryo-EM approach involves flash-freezing the aqueous solution without staining and comprehensively analyzing over 100,000 particles. Thus, it is plausible that the negative-stain EM structure represents a limited subset of the conformational landscape of Keap1. We assume that the cryo-EM results of this study more accurately reflect the native state of Keap1.

Nrf2 inducers are broadly classified into two categories: *i)* electrophilic compounds that target reactive cysteine thiols of Keap1 and *ii)* non-electrophilic PPI inhibitors that disrupt the Keap1-Nrf2 interaction [3]. Importantly, electrophilic Nrf2 inducers retain the potential to react with other biological thiols, such as glutathione, which may lead to off-target effects and cytotoxicity. Therefore, non-electrophilic Keap1-Nrf2 PPI inhibitors are expected to serve as lower toxicity alternatives for Nrf2 induction and efficacy [[39], [40], [41]]. Our analysis demonstrated that CH7450924 induces dissociation of Keap1-Neh2 interaction at equimolar concentration with Keap1, *i.e.,* Keap1:Neh2:CH7450924 = 2:1:2. Notably, this effect was saturated at a two-fold molar excess of the inhibitor, indicating that CH7450924 is a potent disruptor of the Keap1-Nrf2 interaction. Moreover, CH7450924 has been reported to exhibit potent inhibitory activity against the Keap1-Nrf2 interaction, with a low-nanomolar IC_50_, consistent with its activity as a non-covalent competitive inhibitor [33]. These results indicate that most fraction of the added CH7450924 is associated with Keap1 DC domains under our experimental conditions. Although not examined in the present study, it seems possible and important to apply the present analytical system to clarify whether modifications of reactive cysteine residues by electrophilic Keap1 inhibitors are also accompanied by changes in the inter-DC domain distance.

We found that PEGylation effectively alleviated the preferred orientation of Keap1 particles, consistent with previous reports [35]. In the present study, detergents were added in combination with PEGylation, which may have further reduced orientation bias. Importantly, the inter-DC domain distance distributions were highly similar between PEGylated and non-PEGylated preparations under all examined conditions, including Neh2-bound and inhibitor-treated states. These results support the notion that the changes in the inter-DC domain distance observed upon Neh2 binding and CH7450924 treatment reflect intrinsic structural changes of Keap1 rather than artifacts arising from preferred particle orientation.

Our analyses using Keap1 deletion mutants revealed that three regions of Keap1, *i.e.*, the N-terminal, C-terminal, and IVR-DC loop regions, significantly influence the structural flexibility of the Keap1 dimer. These findings indicate that these regions contribute to the flexibility of Keap1 and support our hypothesis that the plasticity of these regions prevents high-resolution structural determination of Keap1. We previously demonstrated that three cysteine residues, Cys613, 622, and 624 in the C-terminal region of Keap1 are important for sensing hydrogen peroxide and for suppression of ubiquitin E3 ligase activity [42]. Through forming disulfide bonds with Cys226, these cysteine residues are assumed to induce a conformational change in Keap1 and loss-of-function. In this context, our current results suggest that the conformational change of the C-terminal region via disulfide bond formation regulates the Keap1 function.

Unexpectedly, deletion of the N-terminal region exerted a strong effect on the inter-DC domain distance. Although the precise function of the Keap1 N-terminal region remains unclear, our findings suggest that this region plays a previously unappreciated role in the structure and/or function of Keap1. Likewise, AlphaFold2 structural predictions suggest that deletion of the IVR-DC loop region directly alters the inter-DC domain distance. While our data demonstrate that conformational changes involving the IVR-DC loop are indispensable for Keap1 function, the detailed structural and mechanistic basis of its contributions warrants further investigation.

The dynamic features of the Keap1 structure found in this study are highly consistent with those reported previously for cryo-EM and crystal structures of other BTB family proteins, such as KBTBD4, KBTBD2, KLHL22, and SPOP [[43], [44], [45], [46]]. Among these proteins, KBTBD4, KBTBD2, and KLHL22 share a domain architecture similar to that of Keap1, and their experimentally determined structures closely resemble to the AlphaFold2-predicted structure of human Keap1, supporting the validity of our experimental design [[43], [44], [45]]. Furthermore, cryo-EM analyses of KBTBD4 in both its apo form and its substrate-bound form (UM171–HDAC1–CoREST complex) revealed that the distance between the two DC domains decreases upon substrate binding [43]. A similar conformational change has also been observed in the KLHL22-CUL3-GDH1 (glutamate dehydrogenase I) complex [45]. In contrast, SPOP lacks a DC domain and instead employs an N-terminal MATH domain for substrate recognition. The MATH domain is connected to the BTB domain through a flexible linker, and this flexibility is critical for efficient substrate recognition [46]. Taken together, these findings indicate that BTB family proteins utilize conformational dynamics to facilitate efficient substrate binding.

In conclusion, our study establishes that the conformational change of Keap1 is important for its interaction with Nrf2. Analyses of deletion mutants support the model that Keap1 ubiquitin ligase activity toward Nrf2 is governed by structural changes within the Keap1 dimer. Notably, all three flexible regions, *i.e.*, the N-terminal, C-terminal, and IVR-DC loop regions, appear to influence Keap1 activity by stabilizing or perturbing its conformational state. These findings support a model in which Keap1 functions as a molecular scaffold whose structural plasticity underlies both physiological regulation and stress-induced dysregulation.

## 4 Resource availability

### 4.1 Lead contact

Further information and requests for resources should be directed to and will be fulfilled by Masayuki Yamamoto (masayuki.yamamoto.c7@tohoku.ac.jp) and Seizo Koshiba (seizo.koshiba.b3@tohoku.ac.jp).

### 4.2 Materials availability

All unique reagents, except compound and PEGylated Keap1 related samples, generated in this study are available from the Lead Contact without restriction. Compound can be provided to academic researchers via a material transfer agreement (MTA) upon reasonable request. PEGylated Keap1 related samples are not available for distribution due to limited stock, however, detailed synthesis procedures are provided in the Methods section.

## 5 Methods

### 5.1 Bacterial strains

*Escherichia coli* Rosetta 2 (DE3), BL21(DE3) and BL21-Gold (DE3) cells were used for recombinant protein expression. Cells were cultured in LB medium.

### 5.2 Cell lines

Expi293F cells (Thermo Fisher Scientific) were maintained in Expi293 expression medium at 37 °C with 8% CO_2_ under shaking conditions.

### 5.3 Chemical reagents

CH7450924 [4-[3-[2-chloro-4-[(2R,5R)-2,4,5-trimethylpiperazin-1-yl]benzoyl]-2,4-dihydro-1,3-benzoxazin-8-yl]-5-fluoro-2-(3-oxa-8-azabicyclo[3.2.1]octan-8-yl)benzoic acid hydrate] was synthesized by Chugai Pharmaceutical Co., Ltd. (Kanagawa, Japan) [33].

### 5.4 Protein expression and purification

All Keap1 constructs (full-length and deletion constructs) were expressed as His-tag fusion proteins using the pET15b vector (Novagen). *Escherichia coli* Rosetta 2 (DE3) (Novagen) cells were transformed with the resulting plasmids, and were grown at 37 °C in LB medium. Protein expression was induced at an OD_660_ of approximately 0.4 by adding isopropyl-β-d-thiogalactopyranoside (IPTG) to a final concentration of 0.2 mM. The cultures were then incubated at 15 °C for 17 h. Harvested cells were suspended in 50 mM Tris-HCl buffer (pH 8.0) containing 300 mM NaCl, 1 mM TCEP, 1 mM Phenylmethylsulfonyl fluoride (PMSF) and 5% glycerol, and were subsequently lysed by sonication on ice.

After centrifugation, the separated supernatant was purified by affinity chromatography using a Ni Sepharose™ Excel resin (Cytiva) pre-equilibrated with 50 mM Tris-HCl buffer (pH 8.0) containing 300 mM NaCl, 1 mM TCEP, 5 mM imidazole, and 5% glycerol. The eluted protein was dialyzed overnight at 4 °C against the dialysis buffer (50 mM Tris-HCl buffer (pH 8.0) containing 300 mM NaCl, 1 mM TCEP and 5% glycerol) using 3.5K MWCO dialysis membrane (Slide-A-Lyzer™ MINI Dialysis Devices, 3.5K MWCO, Thermo Fisher). The His-tag region was simultaneously cleaved by HRV 3C protease (Takara) during dialysis. Following cleavage, the sample was further purified by size exclusion chromatography using a Superdex200 10/300 GL column (Cytiva) equilibrated with 50 mM Tris-HCl buffer (pH 8.0) containing 300 mM NaCl and 1 mM TCEP.

The Neh2 region of human Nrf2 (residues M1-G98) was cloned into the pMAL-c2X vector (New England Biolabs). *E. coli* BL21-Gold (DE3) cells containing pG-Tf2 (groES-groEL-tig Pzt-1 Tetracyclin Cm 5) (Takara BIO) were transformed with the resulting plasmid and were grown at 37 °C in LB medium. Protein expression was induced at an OD_660_ of approximately 0.4 by adding IPTG to a final concentration of 0.2 mM. The culture was then incubated at 15 °C for 17 h. Harvested cells were suspended in the sonication buffer and were subsequently lysed by sonication on ice. After centrifugation, the separated supernatant was purified by affinity chromatography using an Amylose resin (BioLabs) equilibrated with 50 mM Tris-HCl buffer (pH 8.0) containing 300 mM NaCl and 1 mM TCEP. The eluted protein was incubated overnight at 4 °C with Factor Xa protease (BioLabs) to cleave the MBP tag. Following cleavage, the sample was further purified by ion exchange chromatography using a ResourceQ column (Cytiva) pre-equilibrated with 50 mM Tris-HCl buffer (pH 8.0) containing 50 mM NaCl and 1 mM TCEP. The eluted protein was subsequently purified by size exclusion chromatography using a Superdex200 10/300 GL column (Cytiva) equilibrated with 50 mM Tris-HCl buffer (pH 8.0) containing 300 mM NaCl and 1 mM TCEP.

For the PEGylation trials, Keap1 and Neh2 were prepared as follows. The gene encoding full-length human Keap1 with an N-terminal Strep-8His-GST-HRV3C tag was synthesized (AZENTA) and cloned into a mammalian expression vector. Expi293F cells (Thermo Fisher Scientific) were transfected with the resulting plasmid and were grown for 3 days. Harvested cells were suspended in 50 mM Tris-HCl buffer (pH 8.0) containing 150 mM NaCl, 1 mM TCEP, 2 mM MgCl_2_, cOmplete EDTA-free Protease Inhibitor Cocktail (Roche), Benzonase nuclease (Sigma) and 5% glycerol, and were subsequently lysed by sonication on ice. After centrifugation, the supernatant was purified by affinity chromatography using a Strep-Tactin XT 4Flow high capacity resin (IBA Lifesciences) pre-equilibrated with 50 mM Tris-HCl buffer (pH 8.0) containing 150 mM NaCl, 1 mM TCEP, and 5% glycerol. After the resin was washed thoroughly with the same buffer, the protein was eluted by cleaving the tag with Turbo3C protease (Fujifilm Wako Pure Chemical) on column. Following cleavage, the sample was passed through a Glutathione Sepharose 4B resin (Cytiva), and was further purified by size exclusion chromatography using a Superdex200 10/300 GL column (Cytiva) equilibrated with 50 mM HEPES-NaOH buffer (pH 8.0) containing 300 mM NaCl and 1 mM TCEP.

For Neh2, *E. coli* BL21 (DE3) cells containing the Neh2 plasmid were grown at 37 °C for 2.5 h in Overnight Express Instant TB Medium (Merck), and then incubated overnight at 16 °C. Harvested cells were suspended in 50 mM Tris-HCl buffer (pH 8.0) containing 150 mM NaCl, 1 mM TCEP, 2 mM MgCl_2_, cOmplete EDTA-free Protease Inhibitor Cocktail (Roche), Benzonase nuclease (Sigma) and 5% glycerol, and were subsequently lysed by sonication on ice. After centrifugation, the supernatant was purified by affinity chromatography using an Amylose resin (BioLabs) pre-equilibrated with 50 mM Tris-HCl buffer (pH 8.0) containing 300 mM NaCl and 5% glycerol. The eluted protein was incubated overnight at 4 °C with Factor Xa protease (BioLabs) to cleave the MBP tag. Following cleavage, the sample was further purified by ion exchange chromatography using a CaptoHiRes Q column (Cytiva) pre-equilibrated with 50 mM Tris-HCl buffer (pH 8.0) containing 50 mM NaCl, 1 mM TCEP, and 5% glycerol. The eluted protein was passed through an Amylose resin to remove the uncleaved protein and free MBP tag. Finally, the protein was subjected to size exclusion chromatography using a Superdex75 Increase 10/300 GL column (Cytiva) equilibrated with 50 mM HEPES-NaOH buffer (pH 8.0) containing 300 mM NaCl and 1 mM TCEP.

### 5.5 Grid preparation for cryo-EM and cryo-ET

Full-length or a series of partially deleted Keap1 protein was dissolved in a 50 mM Tris-HCl buffer (pH 8.0), 300 mM NaCl, and 1 mM TCEP. For preparation of the Keap1, Neh2 and CH7450924 mixture, CH7450924 was added to 3.5 μM Keap1 and 2.3 μM Neh2 and incubated overnight at 4 °C. The final concentration of Keap1 and Neh2 was 1.57 μM and 0.78 μM, respectively. The final concentration of CH7450924 was 0.78, 1.57, or 3.14 μM. A 3 μL aliquot of the sample was applied to glow-discharged Quantifoil R1.2/1.3 Cu 200 mesh grids (Quantifoil Micro Tools). The sample was vitrified after blotting for 4 s using a Vitrobot Mark IV System (Thermo Fisher Scientific) with 100% humidity and 4 °C.

### 5.6 Grid preparation for the PEGylated sample

Keap1 (20.5 μM) was prepared either alone or in mixtures containing CH7450924 and/or Neh2 at the following molar ratios: (i) Keap1 only; (ii) Keap1:CH7450924 = 1:1; (iii) Keap1:Neh2 = 2:1; and (iv) Keap1:Neh2:CH7450924 = 2:1:2. CH7450924 and Neh2 were added from 1 mM and 188 μM stock solutions, respectively.

For PEGylation, samples were incubated on ice for 1 h with MS(PEG)8 (Thermo Scientific) (250 mM in DMSO) at a final concentration of 1 mM. The reactions were quenched by adding 1 M Tris-HCl buffer (pH 8.0) to one-tenth of the reaction volume. PEGylation was confirmed by SDS-PAGE analysis, which showed band shifts consistent with the increased molecular weight of the PEGylated proteins. Protein samples were concentrated using Amicon Ultra-0.5 centrifugal filter devices (Merck Millipore). For the PEGylated samples, fluorinated octyl maltoside (FOM) was added at a final concentration of 0.5 mM to alleviate preferred orientation of particles. A 3 μL aliquot of the sample was applied to glow-discharged Quantifoil R1.2/1.3 Cu 300 mesh grids (Quantifoil Micro Tools) and vitrified after blotting using a Vitrobot Mark IV System (Thermo Fisher Scientific) at 4 °C and 90–100% humidity.

### 5.7 Cryo-FIB milling

Cryo-FIB milling was performed using a JIB-4700F FIB-SEM (JEOL), equipped with a Leica Microsystems EM VCT500 cryo stage and cryo transfer system (Leica) [47]. Prior to milling, the grids were sputter coated with a 10 nm layer of platinum using an EM ACE600 (Leica). Subsequently, an organic platinum was applied via gas injection system (GIS) deposition for 60 s using the JIB-4700F. Samples were imaged by SEM (3 kV, 100 pA) and were milled by gallium ion beam (30 kV or 15 kV, 1–30 pA). The final thicknesses of the lamellae were approximately 100 nm.

### 5.8 Cryo-EM/cryo-ET data collection

For non-PEGylated samples, cryo-EM data were collected using a 300 kV cryo-transmission electron microscope, the CRYO ARM™ 300 II (JEOL), equipped with a JEOL in-column Omega energy filter and a K3 BioQuantum detector (GATAN). Movies were automatically acquired using SerialEM [48] v3.7 software at 60,000× nominal magnification (corresponding to a physical pixel size of 0.788 Å) with a total dose of 80 e^−^/Å^2^ and a defocus range of −0.8 μm to −2.0 μm. Cryo-ET data were acquired at 30,000× nominal magnification (1.68 Å/pixel) using PACEtomo [49] scripts within SerialEM software. Each tilt series was acquired as 10-frame movies from −50° to +50° with a 3° tilt increment using the dose-symmetric scheme. The target total dose was 80 e^−^/Å^2^ and the target defocus was −5 μm. A total of 34 tilt-series were used for 3D reconstruction. For FIB-milled thin lamellae, images were acquired at 30,000× nominal magnification with a total dose of 20 e^−^/Å^2^ and a target defocus of −30 μm.

For PEGylated samples, cryo-EM data were collected using a 300 kV cryo-transmission electron microscope, Titan Krios G4 (Thermo Fisher Scientific), equipped with a cold FEG, a Selectris-X energy filter and a Falcon 4i direct electron detector (Thermo Fisher Scientific). Movies were automatically acquired using EPU software at 165,000× nominal magnification (corresponding to a physical pixel size of 0.7265 Å) with a total dose of 60 e^−^/Å^2^ and a defocus range of −1.5 μm to −2.5 μm.

### 5.9 Cryo-EM data processing

All cryo-EM image processing, including motion correction, CTF estimation, particle picking, extraction, and 2D classification, was performed within a single RELION pipeline (v4.0.0) [50]. Movie frames were aligned using MotionCor2, and CTF estimation was performed on the dose-weighted sums using CTFFIND 4.1 [51]. A subset of approximately 100 micrographs was manually curated, and the extracted particles were cleaned via 2D classification to generate high-quality templates. These templates were subsequently used to train Topaz v0.2.5 [52], which then automatically picked the full dataset. The resulting particle coordinates were extracted and were subjected to 2D classification. Classes dominated by ice contamination, aggregates, or poor contrast were discarded. Data collection and processing parameters for the Keap1 and Keap1-Neh2 samples, PEGylated samples and Keap1 deletion mutants are provided in Supplementary Tables S1, S2, and S3, respectively.

### 5.10 Cryo-ET data processing

All cryo-ET image processing was performed using the RELION pipeline (v5.0.0). Movie frames were aligned using MotionCor2, and CTF estimation was performed on the dose-weighted sums using CTFFIND 4.1. Tilt series were reconstructed after alignment with the AreTomo2 software implemented in RELION. The reconstructed data were visualized and were analyzed using 3dmod software.

### 5.11 Distance analysis between DC domains

A template atomic model was matched to the averaged images from 2D classifications using an in-house program. The X-ray crystal structure of the DC domain of Keap1 (PDB ID: 3VNH, chain A; 2.2 Å resolution) was used as a template. The procedure of the template matching is shown in Fig. 1E.

First, 20,000 rotated atomic models were randomly generated, and projected along the Z-axis into a 2D image at 10 Å resolution. Second, matching between the target image and the set of projected template images was performed by exhaustively searching over all possible pixel-wise translations. For each match, both the score *Z*_*cross*_ and the correlation coefficient *cc* were evaluated. The matching step was repeated until no additional domains were detected. Before matching additional regions, the density of the previously template-matched region in the target image was subtracted.

Statistical distributions (relative frequency) describing the geometry of the domains, such as inter-domain distances, were summarized using population-weighted statistics based on the Class Distribution values, because each class in the 2D classification represents a different number of particles. Details of the matching method are provided in the Supplementary information (Supplementary methods).

### 5.12 Structure prediction

A predicted structure of the Keap1 homodimer was generated using ColabFold (v1.5.5) with default parameters [53], which combines the fast homology search of MMseqs2 [54] with AlphaFold2 [36].

## CRediT authorship contribution statement

**Ritsumi Saito:** Writing – review & editing, Writing – original draft, Methodology, Investigation, Formal analysis, Conceptualization. **Takeshi Kawabata:** Writing – review & editing, Writing – original draft, Software, Methodology, Investigation, Formal analysis. **Seisuke Yamashita:** Writing – review & editing, Writing – original draft. **Keisuke Oki:** Writing – review & editing, Writing – original draft, Investigation. **Takashi Fujii:** Writing – review & editing, Writing – original draft, Investigation. **Hiroki Kawauchi:** Investigation. **Yoshiaki Doi:** Supervision. **Machiko Irie:** Supervision. **Takuya Torizawa:** Supervision. **Kengo Kinoshita:** Writing – review & editing. **Masayuki Yamamoto:** Writing – review & editing, Supervision, Funding acquisition. **Seizo Koshiba:** Writing – review & editing, Supervision, Conceptualization.

## Declaration of competing interest

The authors declare the following financial interests/personal relationships which may be considered as potential competing interests: Keisuke Oki, Takashi Fujii, Hiroki Kawauchi, Yoshiaki Doi, Machiko Irie and Takuya Torizawa are employees of Chugai Pharmaceutical Co., Ltd. This research was supported in part by Chugai Pharmaceutical Co., Ltd. The other authors declare that they have no known competing financial interests or personal relationships that could have appeared to influence the work reported in this paper.

## Data Availability

Data will be made available on request.
