# Supplementary material for: Inter-domain distance analysis reveals conformational changes in the Keap1-Nrf2 stress sensor system
Source: Redox Biol. 2026 Sep 1;97:104379. doi: 10.1016/j.redox.2026.104379 (PMC13571941; doi:10.1016/j.redox.2026.104379)
Supplement: Supplementary file 1 [file mmc1.docx]

**Supplementary information**

**Supplementary note 1**

**Particle classification analysis of PEGylated samples**

To evaluate the particle classification of the PEGylated samples, we examined the total number of images and number of particles used for inter-DC domain distance analysis. The ratio of particles for which inter-DC domain distance analysis could be successfully calculated relative to the total number of images used for our inter-domain distance analysis system are shown in Table S1. Intriguingly, whereas inter-DC domain distance can be estimated for most of the total particles of the non-PEGylated Keap1 samples, the ratio of particles whose inter-DC domain distance can be successfully estimated substantially decreased for the PEGylated samples, notably the mixture of Keap1 and Neh2 sample (the ratio is 52%). These results are mainly derived from the increase of only one-globular images in the total number of images for the PEGylated samples (Table S1; italic). Our distance analysis system cannot estimate the inter-DC domain distance of those one-globular images (Figure S1).

We speculate that the increase of one-globular image in the PEGylated sample may reflect one of the following mechanisms. *i*) As a result of the improved preferred orientation, the diversity of the orientation of Keap1 particles within the ice may be significantly increased, which consequently results in enhanced diversity of images derived from Keap1 particles, so that 2D classification algorithm cannot distinguish those images properly, resulting in the increase of poor 2D classification class containing one-globular image. *ii*) The increase of the diversity of Keap1 orientation may result in the elevation of heavily overlapped images of two DC domains. *iii*) PEGylation may decrease the affinity of Keap1 in the homodimer, resulting in the increase of the ratio of Keap1 monomer *iv*) Neh2 contains many lysine residues that can be PEGylated outside the DLGex and ETGE motifs, resulting that the highly PEGylated Neh2 is more soluble and stable than the non-PEGylated Neh2. This suggests that the highly PEGylated Neh2 may be complexed with Keap1 monomer and stabilize Keap1 monomer. As a result, the one globular image derived from Keap1 monomer in complexed with the highly PEGylated Neh2 increases. In addition to one-globular images, a subset of the PEGylated samples contained three-globular images (Fig. S3, Table S4). These images may arise from overlapping neighboring Keap1 dimers, superposition of an additional DC domain from another particle onto a single Keap1 dimer, out-of-plane projections in which densities partially overlap, or local particle association. In such cases, it is difficult to determine which two densities originate from the same Keap1 dimer, and the estimated inter-DC domain distance varies depending on the selected pair of densities. Moreover, these images likely contain densities derived from adjacent particles. Therefore, three-globular images were considered unsuitable for quantitative inter-DC domain distance analysis.

**Supplementary methods**

**Template matching of 3D atomic models onto a 2D image**

Matching 3D atomic models to 2D particle image is an effective way to extract structural information from highly flexible molecules when partial atomic models are available. Although several related studies have been reported previously^1,2^, we developed a template-matching program from scratch to fit multiple structural models onto a single 2D particle image and to obtain distributions of molecular geometry using particle population-weighted statistics.

**Target 2D averaged images and a template 3D atomic model**

The target 2D images were obtained from the file named "class_averages.mrc", which is generated during the 2D classification step in Relion. This file contains a set of averaged 2D images corresponding to each particle class. The atomic model of the Keap1 DC domain (PDB ID: 3VNH; chain A) was pre-rotated so that the three eigenvectors of its covariance matrix align with the X, Y, and Z axes prior to template matching. Since the axis with the smallest variance was assigned to the Z-axis, the axial height direction of the short cylinder-like DC domain was aligned with the Z-axis.

**Template matching of 3D atomic models onto a 2D image**

We developed a custom computer program (“AtmTplMch2D”) implementing a standard template-matching algorithm based on cross-correlation, written in the C programming language. Since a single 2D image can contain more than one DC domain, a procedure for detecting multiple domains was required. This procedure consisted of the following two steps.

***Step 1*: Generation of the projected 2D image from 3D atomic model**: Thousands of projected 2D images were generated from a given 3D atomic model. Each atom in the model was blurred using a Gaussian function with a resolution of 10 Å. The 3D orientations of the projection were randomly generated using Marsaglia’s algorithm^3^. Rotation quaternion **q** was randomly generated as follows:

$$\mathbf{q}=\left[ \begin{matrix} \begin{matrix} x_{1}, & x_{2} \end{matrix}, & \begin{matrix} x_{2}\sqrt{\frac{1-s_{1}}{s_{2}}}, & y_{2}\sqrt{\frac{1-s_{1}}{s_{2}}} \end{matrix} \end{matrix} \right]$$

(1)

where *x*_1_ and *y*_1_ are randomly and uniformly selected in (-1, 1) until *s*_1_ = *x*_1_^2^ + *y*_1_^2^ < 1; similarly, *x*_2_ and *y*_2_ are randomly and uniformly selected in (-1, 1) until *s*_2_ = *x*_2_^2^ + *y*_2_^2^ < 1. The number of random projection directions was set to 20,000, which is close to 22,104, corresponding to the number of directions explored at 10° intervals using the Euler angle representation. For each rotated atomic model, a projected 2D image in the Z-axis orientation was generated.

***Step 2*: matching template images on the target image**: Template matching between the target image and the set of projected template images was performed by exhaustive search over all possible pixel-wise translations. To evaluate each matching by rotation *r* and translation (*u*,*v*), both the score *Z_cross_*(*r,u*,*v*) and the correlation coefficient *cc*(*r*,*u*,*v*) were calculated. The score *Z_cross_*(*r*,*u,v*) indicates the contrast of the matching among all the other possible candidate matches^4^, and the correlation coefficient *cc*(*r*,*u*,*v*) indicates local fitness between the template and the corresponding region of the target image^1,2^.

The cross correlation *cross*(*r,u*,*v*) is defined as follows:

$$cross(r,u,v)=\sum_{(x,y)\in T} t_{r}\left( x,y \right)f(x+u,y+v)$$

(2)

where the *t_r_* is the *r*-th rotated template image and *f* are the target image, respectively. The set *T* is the set of indices for the image *t_r_*. The value of cross correlation *cross*(*r,u*,*v*) is normalized by the *Z_cross_*(*r*,*u,v*) score as follows:

$$Z_{cross}(r,u,v)=\frac{cross\left( r,u,v \right)-E_{U}[cross(r,u,v)]}{\sqrt{V_{U}[cross(r,u,v)]}}$$

(2)

where the set *U* is the set of indices for the all rotations and translations, *E_U_*[] and *V_U_*[] indicate the average and the variance in the set *U*, respectively. The score *Z_cross_*(*r*,*u,v*) indicates the contrast of the matching of the (*u*,*v*)-shifted *r*-th rotation template and the target image *f*, among all the other possible candidate rotations and translations^4^.

The correlation coefficient *cc*(*r,u*,*v*) is defined as follows:

$$cc(r,u,v)=\frac{\sum_{(x,y)\in T} \left[ t_{r}\left( x,y \right)-\bar{t}_{r} \right][f\left( x+u,y+v \right)-\bar{f}(u,v)]}{\sqrt{\sum_{(x,y)\in T} \left[ t_{r}\left( x,y \right)-\bar{t}_{r} \right]^{2}\sum_{(x,y)\in T} \left[ f\left( x+u,y+v \right)-\bar{f}(u,v) \right]^{2}}}$$

(3)

where $\bar{t}_{r}$ is the average density of the image *t_r_*, and $\bar{f}(u,v)$ is the average density of the target image *f* in the (*u*,*v*)-shifted template region. The correlation coefficient indicates local fitness between the template and the corresponding region of the target image *f*^1,2^.

Matches were selected using two criteria. The first criterion is that the correlation coefficient *cc*(*r,u*,*v*) indicating local fitness, is more than the given threshold value *θ*. The value *θ* was set to 0.85 in this study. The second condition is that the score *Z_cross_*(*r*,*u,v*) showing the contrast of the matched region, is more than the threshold *ϕ*. The value *ϕ* was set to 1.8 in this study.

The matching step was repeated until no more domains were detected. Before matching additional regions, the density of the previously template-matched region in the target image should be erased. The densities of template image *f* was subtracted by the linearly scaled target density by the parameters *a* and *b* to generate an erased image $\hat{f}$:

$$\hat{f}\left( x+u,y+v \right)=f\left( x+u,y+v \right)-\left[ a\cdot t_{r}\left( x,y \right)+b \right]$$

(4)

The parameters *a* and *b* are obtained to minimize a following error function *E*(*r*,*u*,*v*) :

$$E(r,u,v)=\frac{1}{2}\sum_{(x,y)\in T} \left[ f\left( x+u,y+u \right)-a\cdot t_{r}\left( x,y \right)-b \right]^{2}$$

(5)

The standard least square fitting procedure can yield the optimal parameter *a* and *b*.

$$\frac{\partial E(r,u,v)}{\partial a}=0, \frac{\partial E(r,u,v)}{\partial b}=0$$

(6)

These equations provide a following parameter *a* and *b* to minimize the error *E*(*r*,*u*,*v*):

$$\left( \begin{matrix} a \\ b \end{matrix} \right)=\left( \begin{matrix} \sum_{\left( x,y \right)\in T} t_{r}^{2}\left( x,y \right) & \sum_{\left( x,y \right)\in T} t_{r}\left( x,y \right) \\ \sum_{\left( x,y \right)\in T} t_{r}\left( x,y \right) & \sum_{\left( x,y \right)\in T} 1 \end{matrix} \right)^{-1}\left( \begin{matrix} \sum_{(x,y)\in T} t_{r}\left( x,y \right)f\left( x+u,y+v \right) \\ \sum_{(x,y)\in T} f\left( x+u,y+v \right) \end{matrix} \right)$$

(7)

**Particle population-weighted statistics of matched template structures**
Since each class in the 2D classification contains a different number of particles, the distributions of geometry of the domains were summarized using particle population-weighted statistics. The proportion of particle images assigned to each class was obtained from the "ClassDistribution" column of the "class_averages.star" file. For example, the relative frequency histogram *h_D_*(*k*) of *k*-th bin for the distance between two domains was calculated as follows:

$$h_{D}(k)=\frac{\sum_{i}^{N_{2}} w_{i}\sigma(D_{k}\leq D\left( \boldsymbol{g}_{i1},\boldsymbol{g}_{i2} \right)<D_{k+1})}{\sum_{i}^{N_{2}} w_{i}}$$

(8)

where *w_i_* is the “ClassDistribution” value for the *i*-th class, the *N*_2_ is the number of class averaged images containing two DC domains, *D*(***g****_i_*_1_, ***g****_i_*_2_) is the distance between center positions ***g****_i_*_1_ and ***g****_i_*_2_ of two DC domains *i*1 and *i*2 in the target image of the *i*-th class, and *D_k_* is the distance for the *k*-th bin, and *σ* (*x*) is a step function which returns 1 only if *x* is true.

**Supplementary figure legends**

**Supplementary Figure S1.**

**Overview of cryo-EM data processing and representative images of Keap1.**

(A) Workflow of data processing for 2D classification. Manually picked particles were used to train Topaz for automated particle picking, followed by automated particle picking using the trained model. Extracted particles were subjected to iterative 2D classification for particle selection, and selected particles were used for inter-domain distance analysis. The number of particles at each step is summarized in Tables S1–S3. This workflow applies to the analyses shown in Figures 2, 3, 5, 7, and 8.

(B) Representative cryo-EM micrograph of Keap1 alone. Scale bar (bottom right): 50 nm.

(C) Representative 2D class averages obtained after 2D classification of the particle images. Scale bar (bottom right): 50 Å.

**Supplementary Figure S2.**

**Purification of Keap1 and Neh2.**

(A) Representative size-exclusion chromatography profile of Keap1 and SDS-PAGE of the peak fraction shown in inset.

(B) Representative size-exclusion chromatography profile of Neh2 and SDS-PAGE of the peak fraction shown in inset.

**Supplementary Figure S3.** **Related to Fig. 5.**

**Representative matching results for the 2:1 mixture of PEGylated Keap1 and PEGylated Neh2.**

A total of sixteen images are displayed, organized into four rows (four images per row). The rows are categorized by the number of DC domains recognized by the distance analysis system (ranging from 0 to 3 domains), which is indicated by the numeral on the far left of each row. White circles in the images indicate mask circles with a diameter of 60 pixels. The center of mass of the fitted atomic model must lie within these mask circles. Small circles indicate the positions of the fitted atomic models. Circle colors are scaled according to correlation coefficients, ranging from red (high correlation) through green (intermediate correlation) to blue (low correlation). The range of correlation coefficient values is from 0.85 to 1.00.

**Supplementary Figure S4. Related to Fig. 7.**

**SDS-PAGE of the N-terminal and C-terminal deletion mutants of Keap1.**

(A) Keap1∆N. (B) Keap1∆C, (C) Keap1∆N∆C.

**Supplementary Figure S5. Related to Fig. 8.**
S**DS-PAGE of the Keap1 mutants containing deletions in the IVR–DC loop region.**
(A) Keap1∆loop(312-324). (B) Keap1∆loop(308-326). (C) Keap1∆N∆C ∆loop(312-324). (D) Keap1∆N∆C ∆loop(308-326).

**Supplementary table legends**

**Supplementary Table S1. Related to Fig. 2 and 3.**

All values represent data collection parameters and counts of micrographs or particles at each step of the data processing workflow (see Fig. S1). K, N, and C denote Keap1, Neh2, and CH7450924, respectively, and sample conditions are expressed as molar ratios. Manually picked particles were used to train Topaz for automated particle picking. “2D classes selected” indicates the number of class averages used for training, and “particles for training” indicates particles used to train the Topaz model. “Autopicked” indicates particles automatically picked using the trained Topaz model, and “particles for 2D classification” indicates particles extracted for subsequent 2D classification. “Selected for analysis” indicates the number of particles used for inter-domain distance analysis.

**Supplementary Table S2. Related to Fig. 5.**

All values represent data collection parameters and counts of micrographs or particles at each step of the data processing workflow (see Supplementary Fig. S1). All samples in this table are PEGylated. Manually picked particles were used to train Topaz for automated particle picking. “2D classes selected” indicates the number of class averages used for training, and “particles for training” indicates particles used to train the Topaz model. “Autopicked” indicates particles automatically picked using the trained Topaz model, and “particles for 2D classification” indicates particles extracted for subsequent 2D classification. “Selected for analysis” indicates the number of particles used for inter-domain distance analysis.

**Supplementary Table S3. Related to Fig. 7 and 8.**

All values represent data collection parameters and counts of micrographs or particles at each step of the data processing workflow (see Supplementary Fig. S1). Deletion mutants include N-terminal, C-terminal, and IVR–DC loop deletions of Keap1, as illustrated in Fig. 6B. Manually picked particles were used to train Topaz for automated particle picking. “2D classes selected” indicates the number of class averages used for training, and “particles for training” indicates particles used to train the Topaz model. “Autopicked” indicates particles automatically picked using the trained Topaz model, and “particles for 2D classification” indicates particles extracted for subsequent 2D classification. “Selected for analysis” indicates the number of particles used for inter-domain distance analysis.

**Supplementary Table S4.** **Related to Figure 5.**

Comparison of domain-number distributions successfully identified by template matching. The percentages in parentheses show the proportion of particles relative to the total particle input for the analysis

**Supplementary figures**

**Supplementary Figure S1**

**Supplementary Figure S2**

**Supplementary Figure S3**

**Supplementary Figure S4**

**Supplementary Figure S5**

**Supplementary tables**

**Supplementary Table S1.**

**Data collection and processing parameters for Keap1 and Keap1-Neh2 samples**

|  | K | K:N=2:1 | K:N:C=2:1:1 | K:N:C=2:1:2 | K:N:C=2:1:4 | K:N:C=1:1 |
| --- | --- | --- | --- | --- | --- | --- |
| **Data collection** |  |  |  |  |  |  |
| Magnification | 60,000 | 60,000 | 60,000 | 60,000 | 60,000 | 60,000 |
| Voltage (kV) | 300 | 300 | 300 | 300 | 300 | 300 |
| Electron exposure (e^-^/Å^2^) | 80 | 80 | 80 | 80 | 80 | 80 |
| Defocus range (µm) | -0.8 to -2.0 | -0.8 to -2.0 | -0.8 to -2.0 | -0.8 to -2.0 | -0.8 to -2.0 | -0.8 to -2.0 |
| Pixel size (Å) | 0.788 | 0.788 | 0.788 | 0.788 | 0.788 | 0.788 |
| Micrographs | 4,671 | 4,833 | 4,509 | 4,941 | 4,455 | 4,644 |
| **Topaz training** |  |  |  |  |  |  |
| Manually picked particles | 1,337 | 1,033 | 1,461 | 1,563 | 1,107 | 2,504 |
| 2D classes selected | 3 | 4 | 4 | 5 | 6 | 7 |
| Particles for training | 1,010 | 780 | 748 | 871 | 895 | 2,317 |
| **Topaz autopicking** |  |  |  |  |  |  |
| Autopicked particles | 1,190,692 | 1,633,770 | 1,411,265 | 1,232,964 | 1,414,409 | 717,309 |
| Particles for 2D classification | 297,906 | 342,644 | 548,579 | 404,198 | 499,980 | 358,013 |
| **2D classification** |  |  |  |  |  |  |
| 2D classification rounds | 18 | 13 | 14 | 10 | 9 | 14 |
| Selected particles | 151,370 | 161,619 | 246,466 | 286,857 | 288,515 | 229,448 |

**Supplementary Table S2.**

**Data collection and processing parameters for PEGylated samples**

|  | K | K:N = 2:1 | K:N:CH = 2:1:2 | K:CH = 1:1 |
| --- | --- | --- | --- | --- |
| **Data collection** |  |  |  |  |
| Magnification | 165,000 | 165,000 | 165,000 | 165,000 |
| Voltage (kV) | 300 | 300 | 300 | 300 |
| Electron exposure (e^-^/Å^2^) | 60 | 60 | 60 | 60 |
| Defocus range (µm) | -1.5 to -2.5 | -1.5 to -2.5 | -1.5 to -2.5 | -1.5 to -2.5 |
| Pixel size (Å) | 0.7265 | 0.7265 | 0.7265 | 0.7265 |
| Micrographs | 12,470 | 11,791 | 11,595 | 10,266 |
| **Topaz training** |  |  |  |  |
| Manually picked particles | 904 | 1,035 | 680 | 1,417 |
| 2D classes selected | 9 | 10 | 4 | 5 |
| Particles for training | 823 | 970 | 527 | 538 |
| **Topaz autopicking** |  |  |  |  |
| Autopicked particles | 2,634,424 | 2,640,848 | 2,389,809 | 2,513,071 |
| Particles for 2D classification | 1,336,684 | 1,505,065 | 1,054,724 | 1,100,391 |
| **2D classification** |  |  |  |  |
| 2D classification rounds | 18 | 16 | 21 | 20 |
| Selected particles | 454,812 | 299,478 | 496,765 | 481,719 |

**Supplementary Table S3.**

**Data collection and processing parameters for deletion mutants of Keap1**

|  | K | K:N = 2:1 | ∆N | ∆C | ∆N∆C | ∆loop  (312-324) | ∆loop  (308-326) | ∆N∆C∆loop  (312-324) | ∆N∆C∆loop  (308-326) |
| --- | --- | --- | --- | --- | --- | --- | --- | --- | --- |
| **Data collection** |  |  |  |  |  |  |  |  |  |
| Magnification | 60,000 | 60,000 | 60,000 | 60,000 | 60,000 | 60,000 | 60,000 | 60,000 | 60,000 |
| Voltage (kV) | 300 | 300 | 300 | 300 | 300 | 300 | 300 | 300 | 300 |
| Electron exposure (e^-^/Å^2^) | 80 | 80 | 80 | 80 | 80 | 80 | 80 | 80 | 80 |
| Defocus range (µm) | -0.8 to -2.0 | -0.8 to -2.0 | -0.8 to -2.0 | -0.8 to -2.0 | -0.8 to -2.0 | -0.8 to -2.0 | -0.8 to -2.0 | -0.8 to -2.0 | -0.8 to -2.0 |
| Pixel size (Å) | 0.788 | 0.788 | 0.788 | 0.788 | 0.788 | 0.788 | 0.788 | 0.788 | 0.788 |
| Micrographs | 6,183 | 4,374 | 8,313 | 6,021 | 4,053 | 4,617 | 5,022 | 5,015 | 4,569 |
| **Topaz training** |  |  |  |  |  |  |  |  |  |
| Manually picked particles | 1,186 | 1,519 | 960 | 1,014 | 847 | 1,007 | 737 | 873 | 792 |
| 2D classes selected | 3 | 5 | 7 | 7 | 11 | 3 | 5 | 4 | 4 |
| Particles for training | 834 | 1,187 | 932 | 999 | 825 | 984 | 601 | 822 | 705 |
| **Topaz autopicking** |  |  |  |  |  |  |  |  |  |
| Autopicked particles | 1,872,018 | 1,131,496 | 3,040,886 | 2,229,446 | 1,478,966 | 2,502,501 | 1,579,725 | 2,376,069 | 2,072,130 |
| Particles for 2D classification | 1,038,971 | 490,469 | 903,527 | 1,030,292 | 617,287 | 837,947 | 742,712 | 876,079 | 763,005 |
| **2D classification** |  |  |  |  |  |  |  |  |  |
| 2D classification rounds | 11 | 16 | 10 | 10 | 10 | 11 | 10 | 10 | 10 |
| Selected particles | 320,771 | 273,636 | 739,716 | 684,971 | 503,521 | 822,208 | 415,341 | 722,023 | 632,066 |

**Supplementary Table S4
Comparison of domain-number distributions successfully identified**

|  | Input | 0 | 1 | **2** | 3 |
| --- | --- | --- | --- | --- | --- |
| Keap1 free PEG(-) | 149,623 | 0 (0%) | 0 (0%) | **149,623 (100%)** | 0 (0%) |
| Keap1 + Neh2 PEG(-) | 158,962 | 0 (0%) | 0 (0%) | **142,893 (89.9%)** | 16,069 (10.1%) |
| Keap1 free PEG(+) | 449,094 | 0 (0%) | *93,644 (20.9%)* | **353,023 (78.6%)** | 2,427 (0.5%) |
| Keap1 + Neh2 PEG(+) | 293,493 | 9,580 (3.3%) | *122,216 (41.6%)* | **152,238 (51.9%)** | 9,459 (3.2%) |

**Supplementary references**

1.	Velázquez-Muriel, J., Lasker, K., Russel, D., Phillips, J., Webb, B.M., Schneidman-Duhovny, D., and Sali, A. (2012). Assembly of macromolecular complexes by satisfaction of spatial restraints from electron microscopy images. Proceedings of the National Academy of Sciences *109*, 18821–18826. https://doi.org/10.1073/pnas.1216549109.

2.	Matsumoto, A., Miyazaki, N., Takagi, J., and Iwasaki, K. (2017). 2D hybrid analysis: Approach for building three-dimensional atomic model by electron microscopy image matching. Sci. Rep. *7*, 377. https://doi.org/10.1038/s41598-017-00337-y.

3.	Marsaglia, G. (1972). Choosing a Point from the Surface of a Sphere. The Annals of Mathematical Statistics *43*, 645–646. https://doi.org/10.1214/aoms/1177692644.

4.	Cruz-León, S., Majtner, T., Hoffmann, P.C., Kreysing, J.P., Kehl, S., Tuijtel, M.W., Schaefer, S.L., Geißler, K., Beck, M., Turoňová, B., et al. (2024). High-confidence 3D template matching for cryo-electron tomography. Nature Communications 2024 15:1 *15*, 1–14. https://doi.org/10.1038/s41467-024-47839-8.
